# SOD2 Deficient Erythroid Cells Up-Regulate Transferrin Receptor and Down-Regulate Mitochondrial Biogenesis and Metabolism

**DOI:** 10.1371/journal.pone.0016894

**Published:** 2011-02-04

**Authors:** Florent M. Martin, Xiuling Xu, Katharina von Löhneysen, Timothy J. Gilmartin, Jeffrey S. Friedman

**Affiliations:** 1 Department of Molecular and Experimental Medicine, The Scripps Research Institute, La Jolla, California, United States of America; 2 DNA Array Core Facility, The Scripps Research Institute, La Jolla, California, United States of America; Yale Medical School, United States of America

## Abstract

**Background:**

Mice irradiated and reconstituted with hematopoietic cells lacking manganese superoxide dismutase (SOD2) show a persistent hemolytic anemia similar to human sideroblastic anemia (SA), including characteristic intra-mitochondrial iron deposition. SA is primarily an acquired, clonal marrow disorder occurring in individuals over 60 years of age with uncertain etiology.

**Methodology/Principal Findings:**

To define early events in the pathogenesis of this murine model of SA, we compared erythroid differentiation of Sod2^-/-^ and normal bone marrow cells using flow cytometry and gene expression profiling of erythroblasts. The predominant transcriptional differences observed include widespread down-regulation of mitochondrial metabolic pathways and mitochondrial biogenesis. Multiple nuclear encoded subunits of complexes I-IV of the electron transport chain, ATP synthase (complex V), TCA cycle and mitochondrial ribosomal proteins were coordinately down-regulated in Sod2^-/-^ erythroblasts. Despite iron accumulation within mitochondria, we found increased expression of transferrin receptor, *Tfrc*, at both the transcript and protein level in SOD2 deficient cells, suggesting deregulation of iron delivery. Interestingly, there was decreased expression of *ABCb7*, the gene responsible for X-linked hereditary SA with ataxia, a component required for iron-sulfur cluster biogenesis.

**Conclusions/Significance:**

These results indicate that in erythroblasts, mitochondrial oxidative stress reduces expression of multiple nuclear genes encoding components of the respiratory chain, TCA cycle and mitochondrial protein synthesis. An additional target of particular relevance for SA is iron:sulfur cluster biosynthesis. By decreasing transcription of components of cluster synthesis machinery, both iron utilization and regulation of iron uptake are impacted, contributing to the sideroblastic phenotype.

## Introduction

Reconstitution of mice with hematopoietic stem cells deficient in manganese superoxide dismutase (SOD2) results in a hemolytic anemia that recapitulates many features seen in patients with sideroblastic anemia (SA) [1]. SA's are a heterogeneous group of inherited and acquired erythropoietic disorders with defining features being the presence of bone marrow ringed sideroblasts (RS), abnormal erythroblasts with pathologic mitochondrial iron deposition, and impaired heme biosynthesis [2], [3], [4], [5].

The molecular genetic basis for several of the inherited forms of SA has been described. There are two X-linked sideroblastic anemias (XLSAs), one caused by mutations of an erythroid-specific form of the heme biosynthetic enzyme aminolevulinic acid-synthase (*ALAS2*) [6], and one caused by mutation of a mitochondria-localized transport protein, ATP-binding cassette, member b7 (*ABCb7*) [7]—that plays a role in maturation/transport of iron-sulfur cluster containing proteins. Mutation of the pseudouridylate synthase 1 gene (*PUS1*) was found to cause mitochondrial myopathy and SA [8], [9]. How this lesion specifically affects mitochondrial function or iron metabolism is unclear—but it has been proposed that pseudouridylation of small RNA's distinct from tRNA may affect mitochondrial function [10]. Another type of SA presenting in infancy or childhood, Pearson marrow–pancreas syndrome, results from a large deletion in mitochondrial DNA (mtDNA) [11] and evolves features similar to the multi-system mitochondrial disorder Kearn-Sayers syndrome. A single patient has been reported with a splice site mutation in the glutaredoxin 5 gene (*GLRX5*) as a cause of SA [12]. Most recently, mutations in a mitochondrial carrier family gene (*SLC25A38*) have been identified in cases of hereditary SA [13]. Analysis of *SLC25A38* in a larger collection of hereditary SA samples confirmed that this gene is involved in a significant fraction (∼15%) of hereditary SA cases, although a large number of cases remain genetically undefined (>40%) [14].

The majority of cases of SA are acquired, occur in the context of the bone marrow failure syndrome myelodysplasia (MDS) and remain idiopathic. In the small number of acquired SA cases where a defined genetic lesion has been identified, mitochondria are also implicated in pathogenesis. Somatic point mutations of mtDNA encoding subunit 1 of cytochrome c oxidase (COX1; *i.e.*, complex IV of the respiratory chain) have been found in patients with acquired idiopathic sideroblastic anemia (AISA) resulting in measurable respiratory chain dysfunction [15]. There are reports of mutations affecting other portions of the mtDNA including cytochrome b or mitochondrial encoded tRNAs in isolated cases of SA [16], [17], [18], [19]. However, there remains considerable uncertainty as to the role of acquired mtDNA mutations in SA and more broadly in the pathogenesis of MDS [20].

Some cases of acquired, reversible SA have been associated with specific drugs; the antibiotic chloramphenicol exerts its toxicity *via* inhibition of mitochondrial protein synthesis, suppressing the bone marrow, and can induce SA [21]; anti-tuberculosis therapy interferes with pyridoxine metabolism, a cofactor for ALAS2, and can result in SA [22], [23]. Thus, both genetics and drug toxicity data indicate that mitochondrial dysfunction plays a key role in the etiology of SA. While specific mechanisms responsible for iron overload in developing red cells remain poorly understood, mitochondrial dysfunction (due to a diverse array of genetic lesions or toxic exposures) appears to be both necessary and sufficient to create a convergent phenotype of pathologic intra-mitochondrial iron deposition that is characteristic of SA.

SOD2 is a mitochondrial antioxidant enzyme that detoxifies superoxide anion radicals (O_2_^.-^), a byproduct of mitochondrial respiration. SOD2-deficiency induces severe mitochondrial dysfunction in neurons and muscle leading to embryonic or neonatal lethality in homozygous mutant mice [24]. To analyze the impact of SOD2-deficiency in hematopoietic tissues over long periods of time, we have used fetal liver cells as a source of hematopoietic stem cells (HSC) to reconstitute lethally irradiated mice [1]. Sod2^-/-^ HSC rescue irradiated wild-type recipients, however, Sod2^-/-^ chimeric mice have a persistent hemolytic anemia. Sod2^-/-^ red blood cells (RBC) show increased oxidative damage and have a shortened lifespan [1]. Siderocytes (iron-loaded reticulocytes found in marrow or peripheral blood of Sod2^-/-^ chimeric animals) produced enhanced levels of reactive oxygen species (ROS) and showed increased protein oxidative damage, increased mitochondrial number and mass as well as mitochondrial hyperpolarization [25], [26]. These results suggest that intramitochondrial oxidative stress is an important element in the pathogenesis of SA, and is sufficient to induce pathologic iron accumulation in mitochondria of developing RBC [27]. Characterization of sideroblasts isolated from the marrows of patients with acquired or hereditary SA also showed increased ROS production, oxidative damage to protein and mitochondrial hyperpolarization—indicating that these features are shared between the murine SOD2 model and clinical samples [28]. In order to better characterize this unique sideroblastic-like phenotype and to identify putative targets of oxidative stress that may be important regulators of red cell development or iron homeostasis, we compared erythroid development and gene expression patterns between normal and Sod2^-/-^ erythroid progenitors.

## Results

### Sod2^-/-^ erythropoiesis shows a maturational left shift

**Figure 1:**
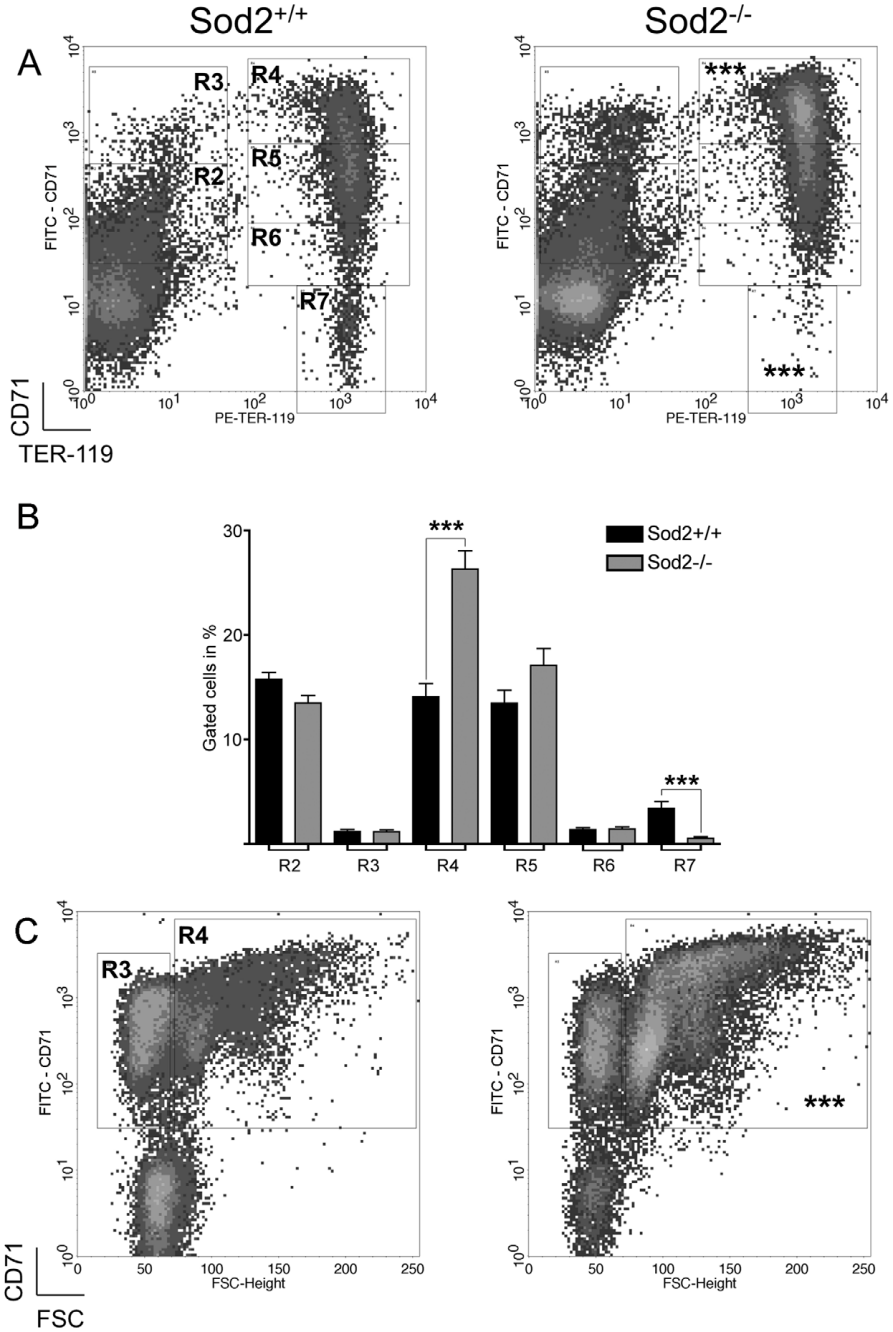
Sod2^-/-^ erythroid development is characterized by a maturational left shift. **A**. Sod2^-/-^ marrow (right) shows an increased fraction of immature basophilic erythroblasts (R4 gate: 1.9-fold increase) and decreased numbers of reticulocytes and mature RBC (R7 gate: 6-fold decrease), respectively, relative to Sod2^+/+^ (left). Density dot plots, gated on FSC^Med-Hi^ PI^-^ cells, allowed the quantitative assessment of the maturational stages of differentiating erythroblasts. Region definitions (clockwise): CD71^Med^ TER-119^Lo^ cells or primitive progenitor cells and proerythroblasts (R2), CD71^Hi^ TER-119^Lo^ cells or proerythroblasts and early basophilic erythroblasts (R3), CD71^Hi^ TER-119^Hi^ cells or early and late basophilic erythroblasts (R4), CD71^Med^ TER-119^Hi^ chromatophilic and orthochromatophilic erythroblasts (R5), CD71^Lo^ TER-119^Hi^ cells or late chromatophilic erythroblasts and reticulocytes (R6), and CD71^-^ TER-119^+^ reticulocytes and mature RBC (R7) (from [29], [30]). **B.** Bar graph comparing the distribution of Sod2^+/+^ and Sod2^-/-^ erythroid progenitor cells in the regions defined above in panel A. Significant differences between groups were observed for: R4, 1.9 fold increase in Sod2^-/-^ (26.33±1.70% of gated Sod2^-/-^ [N = 20] *vs.* 14.10±1.20% Sod2^+/+^ [N = 17] cells; P<0.0001; % gated cells ± SEM); R7, 5.9 fold decrease in Sod2^-/-^ (0.57±0.14% of gated cells *vs.* 3.39±0.63%, respectively; P<0.0001; % gated cells ± SEM; N =  same as above). **C.** Dot plots show the FACS gating strategy used to isolate FSC^Med-Hi^ TER-119^+^ CD71^+^ erythroblast populations (R4) for gene expression analysis. Sod2^-/-^ marrow showed erythroid hyperplasia with a 1.6 fold higher number of erythroid progenitors (R4). Dot plots were gated on PI^-^ TER-119^+^ events and allowed the discrimination of three developmental stages: FSC^Med-Hi^ TER-119^+^ CD71^+^ erythroblasts (R4), FSC^Lo^ TER-119^+^ CD71^+^ reticulocytes (R3) and FSC^Lo^ TER-119^+^ CD71^-^ mature RBC (non-gated on figure). A, B; ***, P<0.0001; Sod2^+/+^, N = 17; Sod2^-/-^, N = 20. FACS profiles shown are representative of multiple assays.

We used a FACS-based method tracking expression of the erythroid-specific antigen TER-119 and CD71 (transferrin receptor) [29], [30] to compare normal and Sod2^-/-^ erythroid differentiation (figure 1A) in murine bone marrow. Taken as a whole, the CD71^Lo-Hi^ TER-119^Hi^ population encompassing the early basophilic erythroblast to reticulocyte stages (gates R4-R6, figure 1A) was increased in Sod2^-/-^ marrows. In particular, CD71^Hi^ TER-119^Hi^ Sod2^-/-^ basophilic erythroblasts (R4) were increased relative to normal (1.9-fold), and Sod2^-/-^ CD71^-^ TER-119^Hi^ reticulocytes and mature RBC (R7) were decreased relative (5.9-fold) to normal (bar graph, figure 1B). Sod2^-/-^ marrow showed a mild erythroid hyperplasia with a 1.6-fold increase in numbers of total erythroblasts (Gate R4 in figure 1C: FSC^Med-Hi^ PI^-^ TER-119^+^ CD71^+^ cells) compared to normal marrow, consistent with prior results [1].

### Microarray-based differential gene expression study of Sod2^-/-^ erythroblasts

In order to better understand functional consequences of loss of SOD2 during erythropoiesis, we identified gene transcriptional changes in erythroid progenitor cells using cDNA microarray-based gene expression analysis. Erythroblasts were isolated from marrow of reconstituted mice using a sorting gate (figure 1C region R4) that includes regions R4-, R5- and R6-gated events (from figure 1A) encompassing all developing erythroid cells co-expressing TER-119 and CD71 for the preparation of total RNA. By genotype, sorted cells did not show a significant difference in cell size or morphology upon microscopic examination. Total RNA (n = 4 *per* genotype) was labeled and hybridized on Affymetrix murine arrays to determine relative gene expression between normal and Sod2^-/-^ erythroblasts.

### Analysis of gene expression data

**Figure 2:**
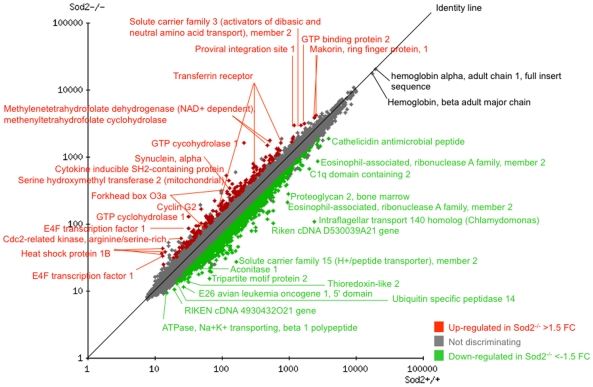
Overview of gene expression comparison of Sod2^-/-^ *vs.* Sod2^+/+^ erythroblasts. Scatterplot highlights genes expressed >1.5 fold higher (red) or lower (green) in Sod2^-/-^ compared to Sod2^+/+^ cells. X and Y axes plot relative expression values derived from microarray intensities. Spots corresponding to selected highly differentially expressed genes are identified. Among the most highly expressed genes are the hemoglobin alpha and beta chains, that show no difference between Sod2^-/-^ and Sod2^+/+^ samples. In Sod2^-/-^ erythroblasts, there are more than twice as many transcripts with reduced vs. increased expression. A full listing of differential transcripts (with corrected p<0.05 and fold-change >±1.5) is in table S1.

The GeneSifter program was used to identify and categorize differentially expressed transcripts. Among the 476 differentially expressed transcripts (identified using a minimum fold change of ±1.5 and corrected p value <0.05) 149 were expressed at higher levels in Sod2^-/-^ erythroblasts, while 327 were down-regulated. A scatterplot of comparative gene expression, with selected differentially expressed transcripts identified, is shown in figure 2. The most highly differentially down- and up-regulated transcripts (showing a fold-change ≥±2) are listed in tables 1 (down) and 2 (up). The entire dataset of 476 transcripts can be found in supplementary data table S1. Overall there are more than twice as many down-regulated transcripts in the Sod2^-/-^ samples.

**Table 1:** List of Expressed Erythroblast Transcripts/Genes with Decreased Expression in Sod2+/+ Compared to Sod2-/- Cells.

Fold Change	Direction in Sod2^+/+^	Adjustedp-value	Gene ID	Gene Name	Gene Identifier	UG Cluster
6.24	Down	0.0134	Gch1	GTP cyclohydrolase 1	NM_008102	Mm.10651
3.48	Down	0.0241	2410003J06Rik	RIKEN cDNA 2410003J06	AK010362	Mm.379207
3.18	Down	0.0347	Hspa1b	Heat shock protein (hsp68)	M12573	Mm.372314
2.88	Down	0.0399	Mthfd2	Methylenetetrahydrofolate dehydrogenase	BG076333	Mm.443
2.85	Down	0.0134	-	ESTs	BB450769	-
2.73	Down	0.0090	E4f1	Transcription factor phi AP3	NM_007893	Mm.163132
2.68	Down	0.0048	-	Similar to E4F transcription factor 1	BB027397	Mm.445985
2.66	Down	0.0129	-	Transcribed locus	BM201499	Mm.450309
2.59	Down	0.0136	-	ESTs	BM247240	-
2.36	Down	0.0217	-	Transcribed locus	BM211430	Mm.413311
2.35	Down	0.0170	-	Transcribed locus	BB631473	Mm.437948
2.33	Down	0.0422	Plek2	Pleckstrin 2	NM_013738	Mm.103380
2.31	Down	0.0170	Epb4.1	Erythrocyte protein band 4.1	BB462549	Mm.30038
2.27	Down	0.0129	-	Transcribed locus	BB201490	Mm.445692
2.16	Down	0.0218	-	DNA segment, Chr 5, ERATO Doi 255, expressed	C79489	-
2.16	Down	0.0391	-	Transcribed locus	BE631223	Mm.472907
2.15	Down	0.0240	-	DNA segment, Chr 10, ERATO Doi 276, expressed	BG066654	-
2.13	Down	0.0182	-	Transcribed locus	BF461324	Mm.449557
2.12	Down	0.0129	-	ESTs	BB205419	-
2.09	Down	0.0324	Usp32	Ubiquitin specific peptidase 32	BG071170	Mm.178524
2.05	Down	0.0331	Cbfa2t3	CBF, runt domain, alpha subunit 2, translocated to, 3	NM_009824	Mm.194339
2.04	Down	0.0428	-	Transcribed locus	BB471309	Mm.406286
2.03	Down	0.0347	Malat1	Metastasis associated lung adeno-Ca transcript 1	AK020483	Mm.298256
2.03	Down	0.0397	-	Transcribed locus	BM240223	Mm.438431
2.02	Down	0.0170	Clcn3	Chloride channel 3, transcript variant a,	BB328803	Mm.259751
2.01	Down	0.0399	Per1	Period homolog 1	AF022992	Mm.7373
2	Down	0.0397	D930015E06Rik	RIKEN cDNA D930015E06	BB547420	Mm.28838

(Fold Change ≥2 and corrected p<0.05.).

**Table 2:** List of Expressed Erythroblast Transcripts/Genes with Increased Expression in Sod2+/+ Compared to Sod2-/- Cells.

Fold Change	Direction in Sod2^+/+^	Adjustedp-value	Gene ID	Gene Name	Gene Identifier	UG Cluster
20.11	Up	0.0008	Ift140	Intraflagellar transport 140 homolog	NM_134126	Mm.32802
4.46	Up	0.0493	Slc15a2	Solute carrier family 15 (H+/peptide transporter) member 2	NM_021301	Mm.281804
3.96	Up	0.0397	-	Transcribed locus	AW493533	Mm.441111
3.54	Up	0.0129	Unkl	Unkempt-like	AK004898	Mm.267353
3.23	Up	0.0297	Aldh1a7	Aldehyde dehydrogenase family 1, subfamily A7	NM_011921	Mm.14609
3.04	Up	0.0379	-	murine leukemia retrovirus	BG297038	-
2.71	Up	0.0420	-	2,3-cyclic nucleotide 3 phosphodiesterase	BB251922	-
2.7	Up	0.0347	Cd79a	CD79A antigen	NM_007655	Mm.1355
2.55	Up	0.0440	Cdk4	Cyclin-dependent kinase 4	NM_009870	Mm.6839
2.54	Up	0.0391	Spib	Ets transcription factor Spi-B	BM244106	Mm.8012
2.4	Up	0.0411	Selenbp2	Selenium binding protein 2	NM_019414	Mm.225405
2.37	Up	0.0241	Scd1	Strain BALB/c stearoyl-coenzyme A desaturase 1	NM_009127	Mm.267377
2.33	Up	0.0493	Cd79b	CD79B antigen	NM_008339	Mm.2987
2.26	Up	0.0170	Tgtp	T-cell specific GTPase	NM_011579	Mm.15793
2.25	Up	0.0428	-	Transcribed locus	BB534670	Mm.406799
2.13	Up	0.0170	Cnp	2,3-cyclic nucleotide 3 phosphodiesterase	M58045	Mm.15711
2.13	Up	0.0170	Hmgn1	High mobility group nucleosomal binding domain 1	NM_008251	Mm.2756
2.1	Up	0.0347	Msh6	MutS homolog 6	U42190	Mm.18210
2.08	Up	0.0396	Car1	Carbonic anhydrase 1	BC011223	Mm.273195
2.07	Up	0.0315	Ifit1	Interferon-induced protein with tetratricopeptide repeats 1	NM_008331	Mm.439751
2.07	Up	0.0428	2900010J23Rik	RIKEN cDNA 2900010J23	BI144310	Mm.27344
2.06	Up	0.0493	Tmem179b	Transmembrane protein 179B	NM_026325	Mm.45155
2.06	Up	0.0170	Lgals9	Lectin, galactose binding, soluble 9	NM_010708	Mm.341434
2.05	Up	0.0347	C1qbp	Complement component 1, q subcomponent binding protein	NM_007573	Mm.30049
2.05	Up	0.0347	Fam132a	Family with sequence similarity 132, member A	NM_026125	Mm.29140
2.02	Up	0.0312	Hspb6	Heat shock protein, alpha-crystallin-related, B6 (Hspb6)	BB755506	Mm.34885
2	Up	0.0241	Hn1l	Hematological and neurological expressed 1-like	AV116987	Mm.371601

(Fold Change ≥2 and corrected p<0.05.).

**Figure 3:**
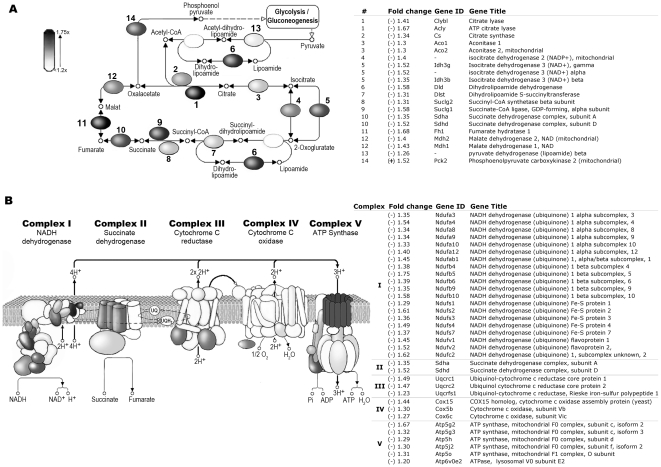
Sod2^-/-^ cells show decreased expression of multiple components of the TCA cycle and Electron Transport Chain. **A**. Schematic of TCA cycle highlights those enzymes with reduced transcript levels. Degree of shading reflects the degree of transcript expression change. The associated table shows enzyme name and fold change observed. Nearly every enzyme in the pathway shows a statistically significant reduction in transcription, although the fold-change is modest. **B**. Schematic diagram of ETC complexes I-IV and ATP synthase highlights nuclear encoded components with decreased expression in Sod2^-/-^ cells. The accompanying table lists specific subunit names along with the fold change observed. Each complex shows decreased transcription of one or more nuclear encoded subunits. All highlighted subunits in panels A and B showed statistically significant differential expression (p<0.05), with fold-change as indicated in table or by grey-scale.

We used gene ontology/pathway analysis [31] to identify patterns of gene expression change characteristic of specific pathways or biological functions affected by SOD2-deficiency. The largest gene sets identified as significantly altered (with a Z-score >±2) [32] in Sod2^-/-^ erythroblasts comprised metabolic pathways with a direct role in mitochondrial metabolism or biogenesis. Nearly every enzyme in the tricarboxylic acid (TCA) cycle was down-regulated at the transcriptional level (figure 3a). The exception was the enzyme phosphoenolpyruvate carboxykinase 2 (Pck2), a regulator of substrate flow between the TCA cycle and glycolysis/gluconeogenesis. Similarly, loss of SOD2 profoundly effects transcription of multiple nuclear-encoded components of all 5 mitochondrial respiratory chain complexes (figure 3b). Mitochondrial protein synthesis is also targeted, with 42 nuclear encoded ribosomal protein genes coordinately down-regulated in Sod2^-/-^ erythroblasts (figure S1 shows a heat map of mitochondrial ribosomal gene expression; all down-regulated ≥1.2 fold with corrected p≤0.05). Additional pathways that differ between Sod2^+/+^ and Sod2^-/-^ erythroblasts include spliceosome, DNA repair and replication, purine and pyrimidine metabolism and gene sets associated with Huntington's and Parkinson's diseases (supplementary tables S2 and S3 list all pathways that differ between Sod2^+/+^ and Sod2^-/-^ with a Z-score > ±2).

**Figure 4:**
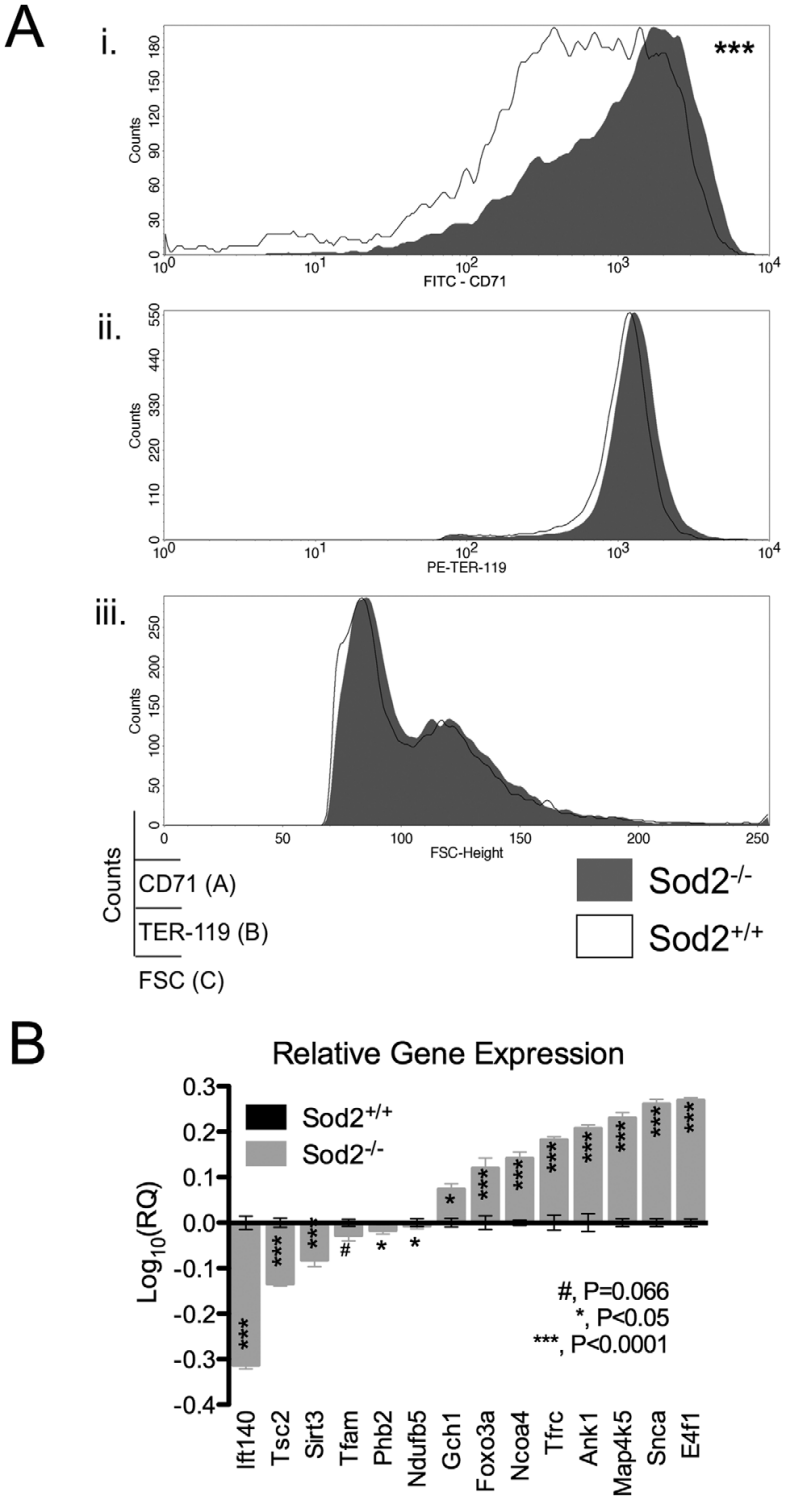
Validation of Microarray Results at Protein Level (CD71) and by qPCR. **A**. Transferrin Receptor Expression: Smoothed histograms excluding dead cells display (**i**) CD71, (**ii**) TER-119, (**iii**) FSC height *vs.* cell counts. Bone marrow cell suspensions were labeled with anti-TER-119, -CD71 or isotype control mAbs plus propidium iodide, and were assayed by FACS. Events were gated on FSC^Med-Hi^ PI^-^ TER-119^+^ cells. Staining specificity was determined by comparison with staining using an isotype control mAb for CD71 (not shown). Sod2^+/+^ and Sod2^-/-^ cells express similar levels of TER-119 antigen (1319±145.30 *vs.* 1352±140.60) and have similar forward scatter profiles (103.80±1.35 *vs.* 106.50±1.64), yet Sod2^-/-^ cells show a 1.6-fold increase in transferrin receptor 1 (CD71) expression (528.80±51.07 *vs.* 851.10±57.29, Sod2^+/+^ *vs.* Sod2^-/-^). This increase appears to represent both a developmental shift (see figure 1A), and an increase in absolute expression within individual cells. Values presented are geometric mean fluorescence intensity (Geo MFI) ± SEM; Sod2^-/-^, N = 20; Sod2^+/+^, N = 17; ***, P<0.0001. **B.** qPCR validation of microarray study: Graph shows the relative expression of 14 transcripts determined by qPCR. Expression levels in Sod2^-/-^ erythroblasts (grey) are presented relative to Sod2^+/+^ samples as calibrator (black); the latter being given an expression level of 1 (Log_10_(1) = 0), Log_10_ transformed and vertically stacked. TaqMan assays were chosen so that they covered a wide range of expression from the most down-regulated to the most up-regulated transcripts in Sod2^-/-^ erythroblasts. Results were normalized to the expression of eukaryotic 18S rRNA as endogenous control. 13/14 transcripts were significantly different between Sod2^+/+^ and Sod2^-/-^ samples. All transcripts showed the expected direction of differential expression based upon microarray results. RQ, relative quantity (X axis); mean ± SEM; N = 4 biological replicates *per* genotype, run in technical triplicates; figure indicates p values from unpaired, 2 tailed t test.

Genes involved in hemoglobin biosynthesis, iron metabolism or identified as known causes of sideroblastic anemia were queried for differential expression. No significant differences were observed for most genes involved in heme biosynthesis including ALAS-2 and alpha and beta globin chains–which as expected were among the most highly expressed transcripts in erythroblasts (figure 2). A single transcript (Riken cDNA A230051G13) encoding a putative aminomethyltransferase involved in heme biosynthesis was expressed at higher levels in Sod2^+/+^ cells (see supplemental figure S2). Two genes involved in iron metabolism, transferrin receptor (Tfrc) and ATP binding cassette family member b7 (ABCb7) were differentially expressed. *Tfrc*, the cellular gatekeeper for iron, was expressed at higher levels in Sod2^-/-^ cells (figure 4) at both the mRNA and protein levels. *ABCb7*, previously shown to be mutated in X-linked sideroblastic anemia with ataxia (XLSA/A—OMIM 301310) is decreased in Sod2^-/-^ cells (1.6 fold decrease, p = 0.02, unpaired t test). No other genes associated with iron metabolism, heme biosynthesis or hereditary sideroblastic anemia showed significantly different expression between groups. (supplemental figure S2 shows expression heat maps and the list of genes from these analyses).

### Microarray validation at the transcript and protein levels

Expression at the gene and protein level of CD71, transferrin receptor, was of particular interest as SOD2 deficient cells, similar to sideroblasts in patients with SA, show iron overload in the presence of an obvious defect in iron utilization. The higher expression of *Tfrc* found in Sod2^-/-^ erythroblasts was verified both by FACS analysis of CD71 surface expression on bone marrow cell suspensions (figure 4a) and by qPCR (figure 4b). Increased expression of CD71 was not due to differences in cell size, as normal and SOD2 deficient erythroid cells showed similar forward scatter profiles and expressed comparable amounts of the Ter119 surface antigen, yet showed a 1.6 fold increase in transferrin receptor 1 (CD71) surface expression, when comparing geometric means of fluorescence intensity values.

Array data were validated by performing TaqMan Gene Expression Assays on total RNA samples from independently prepared TER-119^+^ CD71^+^ erythroblasts. 13/14 transcripts tested by qPCR showed significant differential expression between Sod2^-/-^ and Sod2^+/+^ samples (2 with p<0.05, 11 with p<0.0001) and all transcripts showed the expected direction of change when comparing Sod2^-/-^ and Sod2^+/+^ cells (figure 4b). The validation set included genes with high, intermediate and low expression levels, as well as the most highly up and down regulated transcripts. Among the validation set are genes with highly differential expression, but no previously described role in red cell development or physiology—such as synuclein α *Snca*–2 fold up, intraflagellar transport protein 140 (*Ift140*—20 fold down), GTP cyclohydrolase (*Gch1*—6 fold up) and tuberous sclerosis 2 (*Tsc2*—2 fold down).

## Discussion

In this study we have evaluated gene expression changes in erythroblasts that result from knockout of the antioxidant protein SOD2 in hematopoietic tissues—a model previously shown to have many features of congenital or acquired sideroblastic anemia. Comparative FACS analysis using erythroid specific markers (Ter119 and CD71—figure 1) demonstrates a maturational shift in SOD2 deficient marrow, with more immature cells, particularly basophilic erythroblasts, relative to wild-type marrows. This is consistent with prior work showing erythroid hyperplasia in spleen and marrow of recipients of Sod2^-/-^ cells [1].

SOD2-deficiency had profound effects on transcription of genes involved in mitochondrial metabolism, with multiple transcripts encoding proteins involved in oxidative phosphorylation, ATP synthesis, TCA cycle and mitochondrial biogenesis down-regulated in Sod2^-/-^ erythroblasts (figure 3). An analogous global decline in activity was found when measuring mitochondrial respiratory complex activity in tissues of Sod2^-/-^ neonates prior to death [24], [33]. In particular, nuclear-encoded components of all 5 mitochondrial respiratory complexes showed reduced transcript levels, as did multiple enzymes in the TCA cycle. An exception was the TCA cycle enzyme phosphoenolpyruvate carboxykinase 2 (Pck), that showed an increase in expression in SOD2 deficient cells. Pck converts the TCA cycle anion oxaloacetate to phosphoenolpyruvate, which can in turn be converted to pyruvate by pyruvate kinase (PK) or to glucose. Increased expression of Pck2 suggests that substrates are being shunted away from the TCA cycle toward glycolysis/gluconeogenesis in Sod2^-/-^ cells [34].

A major mechanism underlying the development of mitochondria-related diseases is thought to be an increase in intracellular oxidative stress resulting from impairment of the mitochondrial electron transport chain (ETC) [35], [36]. Our microarray results show that in the context of SOD2 deficiency, oxidant stress leads to a broad down-regulation of genes encoding components of the respiratory chain. This may be due to a direct effect of superoxide on a signaling pathway between the nucleus and mitochondria, or may result from a decline in the function of oxidant sensitive components of the electron transport chain (such as Fe:S containing proteins)—resulting in a metabolic imbalance that triggers mitochondrial-to-nuclear communication.

Widespread down regulation of expression of nuclear encoded mitochondrial genes/proteins suggests engagement of a feedback loop controlling transcription of nuclear-encoded mitochondrial proteins. Candidate components of such a feedback loop include the related nuclear coactivators PGC1α, PGC1β and PPRC1, which regulate expression of many proteins involved in oxidative phosphorylation and mitochondrial biogenesis [37], [38], [39], including many genes in the set of down regulated messages in Sod2^-/-^ erythroblasts. A prior study investigating erythroid development from hematopoietic stem cells deficient in the tumor suppressor Rb implicated PGC1β as a regulator of erythroid development [40]. PGC1α and PGC1β transcript levels were very low in erythroblasts and did not differ between normal and Sod2^-/-^ cells either in microarray or subsequent qPCR experiments (data not shown). Expression of the related coactivator, PPRC1 [37] was both higher, and differential when comparing Sod2^-/-^ and normal erythroblasts on microarray (1.6 fold > in Sod2^+/+^ vs. Sod2^-/-^, P<0.05) implicating PPRC1 as a candidate regulator of mitochondrial biogenesis during erythroid development. While microarray analysis does not assess transcription of mitochondrial DNA-encoded subunits of the respiratory chain, we also found reduced expression (1.8-fold, with adjusted P<0.02) of the critical nuclear encoded mitochondrial transcription factor A (*Tfam*) in Sod2^-/-^ erythroblasts. *Tfam* is required for the expression of mitochondrial DNA-encoded genes, and is itself a target of PGC1α [41] and, by analogy, a potential target of related family members PGC1β and PPRC1. One prediction that follows from decreased expression of Tfam would be a global decrease in expression of mitochondrial encoded protein subunits of the ETC in SOD2 deficient cells. Also consistent with the theme of decreased mitochondrial biogenesis is the statistically significant, but modest (1.2 to 1.6 fold) decrease in transcription of multiple nuclear encoded mitochondrial ribosomal protein genes in Sod2^-/-^ cells (supplementary figure S1).

Like human sideroblasts, SOD2 deficient erythroblasts do not effectively utilize iron creating a paradoxical situation in which cells are functionally iron-deficient despite (intramitochondrial) iron overload. Microarray data revealed two differentially expressed transcripts involved in iron homeostasis, *Tfrc*–encoding transferrin receptor 1–that is expressed at higher levels in Sod2^-/-^ cells (figure 4b), and *ABCb7*—a mitochondrial membrane localized solute transport protein necessary for iron:sulfur cluster biogenesis. Increased expression of *Tfrc* is likely to facilitate the observed dramatic increase in iron content of SOD2 deficient cells by enhancing iron delivery. In both figure 1A and figure 4A, we show increased Tfrc protein expression by flow cytometric assay in SOD2 deficient erythroid progenitors. This increased expression reflects both the over-representation of basophilic erythroblasts (which are CD71 high by definition) in the SOD2 deficient marrow, and an incremental increase of Tfrc expression within Sod2^-/-^ cells at this developmental stage. This incremental increase in Tfrc expression may be mediated by changes in intracellular iron regulatory protein function. Decreased expression of *ABCb7* (down-regulated in SOD2 deficient cells 1.7-fold) may also exert an effect on iron metabolism through a reduction in Fe:S cluster assembly—and thereby impact iron homeostasis by altering the level of iron regulatory protein 1 (IRP1). Under conditions of iron deficiency, IRP1 activity increases as the apo protein (lacking an Fe:S cluster) accumulates. IRP's bind to regulatory sequences in the *Tfrc* mRNA 3′ untranslated region and stabilize the messenger RNA—promoting production of transferrin receptor protein [42], [43], [44] thereby increasing iron delivery. Decreased transcription of *ABCb7* has been reported in CD34^+^ cells isolated from MDS patients with idiopathic acquired SA (RARS), suggesting that ABCb7 is a critical, perhaps necessary target for the derangement of iron metabolism that leads to generation of ringed sideroblasts [45]. Interestingly, we find that *Ciao1* [46], [47], a gene encoding a second protein required for a late step of iron:sulfur cluster assembly, is also down-regulated in Sod2^-/-^ cells (1.6-fold; p<0.0005 unpaired t test). The same argument can be made that reduced Ciao1 activity has potential to alter erythroid iron metabolism by tipping the balance toward generation of the apo protein, IRP1.

At the level of gene expression profiling, the most prominent effect of loss of SOD2 is a broad down-regulation of multiple aspects of mitochondrial metabolism and biogenesis. This is consistent with current knowledge regarding causes of hereditary SA—where mutations affecting proteins that localize to mitochondria, or mitochondrial DNA itself have been shown to be causal. SA is much more common later in life in the context of myelodysplasia, where etiology remains uncertain. Our results demonstrate that mitochondrial dysfunction (of diverse etiology) can be a primary event in development in SA. In Sod2^-/-^ erythroblasts oxidative stress affects erythroid-specific transcriptional mechanism(s) that regulate expression of nuclear encoded mitochondrial proteins. One consequence of such dysfunction is altered iron metabolism secondary to impaired iron:sulfur cluster synthesis resulting from down-regulation of *ABCb7* and *Ciao1*—which impacts both mitochondrial iron utilization and regulation of iron metabolism. Identification of components of the mitochondrial biogenesis pathway active during erythroid differentiation will be an important step toward improving our understanding of the etiology of SA and will provide additional insights into the relationship between mitochondrial dysfunction and development of anemia. Novel insights into pathogenesis of sideroblastic anemia may come from evaluation of the function of additional candidate genes identified as strongly differentially expressed between normal and Sod2^-/-^ erythroblasts, but with no previously defined role in erythroid development. Evaluation of these loci in both hereditary and acquired SA cases that lack mutations in previously identified SA genes may identify novel mutations associated with this disorder.

## Materials and Methods

### Ethics Statement

All animal experiments were conducted under an approved protocol (ARC 06-0339) approved by the TSRI IACUC committee. All work complies with relevant federal and state regulations concerning use of animals.

### Mice and reagents

Female B6 mice reconstituted with Sod2^+/+^ or Sod2^-/-^ HSC were generated as previously described [1]. Hematopoietic chimeric animals were tested for anemia by assaying hematocrit and reticulocyte counts. Marrow was taken from femurs, tibias, pelvic girdles and humerus after Halothane (Halocarbon Laboratories, River Edge, NJ) vapor terminal inhalation. All experiments were conducted under an approved protocol (ARC 06-339) approved by the TSRI IACUC committee. All work complies with relevant federal and state regulations concerning use of animals. All monoclonal antibodies (mAbs) were purchased from Pharmingen (BD Biosciences, San Jose, CA) save a FITC-rat IgG1, κ isotype control mAb (eBioscience, Inc., San Diego, CA). LS & LD MACS columns and MidiMACS separator units were purchased from Miltenyi Biotech (Auburn, CA, USA); and phenol red–free RPMI 1640 media from Invitrogen (Carlsbad, CA, USA). Propidium iodide (PI) and Hybri-Max RBC lysing buffer were purchased from Sigma-Aldrich (St. Louis, MO, USA).

### Erythroblast sorting for microarray

Bone marrow cell suspensions were labeled with a purified anti-CD16/32 mAb for cell surface Fc fragment receptors (FcR) saturation, FITC-conjugated anti-CD71 and PE-conjugated anti-TER-119 mAbs in ice-cold fluorescence-activated cell sorting (FACS) buffer (2 mM EDTA: 0.5% BSA: PBS). Cells were resuspended in sorting buffer (1 mM EDTA: 25 mM HEPES: 1% FCS: 10 µg/ml PI: PBS). Two pairs of Sod2^+/+^ and Sod2^-/-^ samples were sorted in parallel using two FACSDiva and two FACSAria sorters (BD Biosciences). Ter119^+^, CD71^+^ erythroblasts corresponding to gate R4 in Figure 1C were used for microarray analysis.

### Positive selection of TER-119^+^ erythroid progenitors for quantitative real-time RT-PCR (qPCR)

RBC-depleted bone marrow cell suspensions were labeled with a biotinylated TER-119 mAb; TER-119^+^ cells were pulled down with streptavidin MicroBeads (Miltenyi Biotec) according to the manufacturer's instructions. Purity and cell morphology were evaluated by FACS analysis and microscopic examination of Wright-Giemsa-stained sorted cells cytospun onto slides, respectively. Ter-119^+^ cells represented 85.78%±1.6% of magnetically-purified cells from whole marrow suspensions with 81.94%±3.23% viability (N = 7; trypan blue exclusion; not shown). We extracted and average of 8.29 (±1.16) micrograms total RNA from 18.82 (±3.90)×10^6^ purified erythroid progenitors (N = 7; not shown) as material for validation using RT-qPCR reactions.

### FACS analysis

Cell suspensions were labeled with purified anti-CD16/32 (FcR saturation), FITC-conjugated anti-CD71 and PE-conjugated anti-TER-119 mAbs in ice-cold FACS buffer. 10 µg/ml PI was added prior to analysis on a FACSCalibur cytometer running CellQuest Pro (BD Biosciences). Gating distinct stages of erythroid development (figure 1A) was based upon scheme of Socolovsky [29]. Anti-CD71 labeling specificity was validated by the use of a FITC-rat IgG1, κ isotype control mAb (eBioscience, San Diego, CA*).*

### Total RNA extraction from erythroid cells

Total RNA was extracted using the RNeasy Mini kit (Qiagen, Inc., Valencia, CA) accordingly to the manufacturer's suggested protocol for isolation of Total RNA from animal cells. Briefly, we disrupted and lysed 12.65±1.90 million sorted cells (N = 8) in RLT Buffer, homogenized and sheared genomic DNA through centrifugation on QIAshredder spin columns; we performed on-column DNA digestion with the RNase-Free DNase Set (Qiagen) and eluted total RNA in RNase-free water (Sigma-Aldrich).

### Oligonucleotide arrays

All experiments were performed using GeneChip Mouse Genome 430 2.0 and GLYCOv2 oligonucleotide arrays (Affymetrix, Santa Clara, CA), as described at http://www.affymetrix.com/products/arrays/specific/mouse430_2.affx and http://www.functionalglycomics.org/static/consortium/resources/resourcecoree.shtml, respectively. GLYCOv2 oligonucleotide arrays are custom GeneChip arrays designed for the Consortium for Functional Glycomics (CFG, http://www.functionalglycomics.org/).

### RNA target preparation and array processing

#### Labeling

Total RNA from each sample was used to prepare biotinylated target RNA using standard Affymetrix protocol (http://www.affymetrix.com/support/technical/manual/expression_manual.affx) as previously described [48]. Briefly, 5 µg of total RNA was used to generate first-strand cDNA by using a T7-linked oligo(dT) primer. After second-strand synthesis, *in vitro* transcription was performed with biotinylated UTP and CTP (Enzo Life Sciences, Farmingdale, NY). **Hybridization:** the target cRNA generated from each sample was processed as *per* manufacturer's recommendation using a GeneChip Instrument System (Affymetrix). Briefly, spike controls were added to 6.7 µg and 10 µg fragmented cRNA before overnight hybridization on GLYCOv2 and 430 2.0 arrays, respectively. **Post-processing and scanning:** arrays were then washed and stained with streptavidin-phycoerythrin, before being scanned on a ScanArray 3000 (Affymetrix) using default settings and a target intensity of 250 for scaling. **Quality control (QC):** The amount and quality of starting total RNA and cRNA were checked with a ND-1000 Spectrophotometer (NanoDrop Technologies, Inc., Wilmington, DE) and an Agilent Bioanalyzer (Agilent Technologies, Palo Alto, CA), respectively, and/or on formaldehyde-containing agarose gels. After scanning, array images were assessed by eye to confirm scanner alignment and the absence of significant bubbles or scratches on the chip surface. GAPDH and β-actin 3′/5′ ratios were confirmed to be within acceptable limits although one Sod2^-/-^ specimen, showed slight RNA degradation. Gene Sifter analyses performed with and without this 4^th^ Sod2^-/-^ sample yielded similar results, and all figures/tables are derived from a 4×4 chip comparison. BioB spike controls were found to be present on all chips, with BioC, BioD and CreX also present in increasing intensity. When scaled to a target intensity of 250 using the GeneChip Operating Software (GCOS) v1.3.037 algorithm (Affymetrix), scaling factors for all arrays were within acceptable limits (8.218-12.594 for GLYCOv2 chips, 4.089-7.955 for 430 2.0 arrays), as were background (42-68 for GLYCOv2, 48-67 for 430 2.0), Q values and mean intensities. Details of QC measures can be found at https://www.functionalglycomics.org/glycomics/publicdata/microarray.jsp, by clicking on the ‘*Raw Data*’ icon link for microarray experiment ‘*Jeff Friedman 1: Sod2 KO anemic mice*’.

#### Microarray data analysis

Data presented were generated using GeneSifter (VizX Labs, Seattle, WA) based pairwise analysis, RMA-normalized. CEL files (N = 4 Sod2^+/+^; N = 4 Sod2^-/-^) and were filtered upon the following criteria: a ±1.5 or 1.2 ratio cutoff (fold change, F.C.), *t*-test with Benjamini and Hochberg MTC to adjust for false positives, FDR [49], [50] and adjusted P value <0.05. Using these parameters, we found 476 discriminant transcripts at a ±1.5 fold change (supplement **table S1**), and 2565 at a ±1.2 fold change. MIAME-compliant data discussed in this publication has been deposited at the CFG (https://www.functionalglycomics.org/glycomics/publicdata/microarray.jsp; GLYCOv2 arrays), in NCBIs Gene Expression Omnibus (GEO, http://www.ncbi.nlm.nih.gov/geo/) and is accessible through GEO Series accession number GSE8726 (Mouse Genome 430 2.0 arrays). Gene ontology and KEGG pathway analyses were performed in GeneSifter to identify affected pathways. The entire gene expression dataset (unfiltered for threshold or statistical significance) was used to query expression of sets of genes of interest (for example: heme biosynthesis, sideroblastic anemia, iron metabolism as presented in supplementary data).

#### Two-step TaqMan Gene Expression Assays

Total RNA was concentrated with a DNA100 SpeedVac Concentrator (Savant) and reverse transcribed using the High-Capacity cDNA reverse transcription (RT) kit (Applied Biosystems Inc., Foster City, CA). We performed quantitative real-time PCR (qPCR) using 30 ng cDNA template and the TaqMan Gene Expression Master Mix (ABI) in singleplex reactions on a 7900HT Fast Real-Time PCR System running SDS 2.3 (ABI) in default thermal cycling conditions and endpoint data collection. QC and amplification specificity of all targets was confirmed by melting curve analysis. Data analysis was performed using the RQ Manager 1.2 software (ABI) in ΔΔC_t_ relative quantification mode. Eukaryotic 18S rRNA was used as endogenous control; results were expressed relative to Sod2^+/+^ samples as calibrator and being given an expression level of 1 [51]. Average Ct values for both Sod2^-/-^ and control Sod2^+/+^ samples were based on 4 biological replicates; the Ct value for each biological replicate was the average of technical triplicates. Part numbers of the used assays are shown in supplementary data **table S4**.

### Statistical analysis

Unpaired two-tailed *t*-tests were performed using GraphPad Prism (GraphPad Software, San Diego, CA); results are shown as mean ± SEM; replicate numbers (N).

## Supporting Information

Figure S1 **Heat Map of Mitochondrial Ribosomal Protein Gene Expression.** 42 Distinct nuclear encoded mitochondrial ribosomal protein genes are expressed differentially when comparing Sod2^+/+^ and Sod2^-/-^ samples. Data were filtered to display transcripts with ≥1.2 fold change and a corrected p value <0.05. Using these criteria, 43/45 significantly different transcripts are down regulated in Sod2^-/-^ erythroblasts. 3 of the listed genes appear twice and are represented by 2 distinct probe sets.
(DOC)

Figure S2 **Heat Maps Showing Genes Involved in: Iron Ion Homeostatis, Heme Biosynthesis, or Etiology of Sideroblastic Anemia.** Expression of genes annotated as playing a role in iron homeostasis and heme biosynthesis is displayed for both Sod2^+/+^ and Sod2^-/-^ samples without statistical filtering for fold change or significance. When statistical filters were applied to the set of iron homeostasis genes, *ABCb7* and *Tfrc* were the only genes expressed with a fold change ≥1.5 and p value <0.05. *ABCb7* is down in Sod2^-/-^ cells, while *Tfrc* expression is up. Similarly, genes involved in heme biosynthesis are displayed without filtering on the right. When statistical filters were applied, a putative cDNA (Riken A230051G13) with a proposed role in glycine catabolism and heme biosynthesis was found to be expressed at higher levels in Sod2^+/+^ cells, meeting the same fold-change and statistical criteria. Finally, genes previously identified as mutated in hereditary sideroblastic anemia were queried for expression. Of these genes, only *ABCb7* was (again) found to be significantly differentially expressed. *Sod2* appears in the list of iron homeostasis related genes without showing differential expression. This reflects detection of expressed (but deleted for exon 3, and therefore nonfunctional [24]) mRNA in the Sod2-/- cells, as some of the probesets on the microarray for detecting this gene are outside of the deleted exons.
(DOC)

Table S1 **List of 476 Differentially Expressed Transcripts Comparing Sod2^-/-^ and Sod2^+/+^ Erythroblast Samples.** Criteria used in filtering data were fold change ±1.5 with a corrected p value <0.05 (Benjamini and Hochberg MTC used).
(DOC)

Table S2 **KEGG Pathway Analysis of the 476 most highly differentially expressed genes.** The GeneSifter program was used to generate a list of KEGG pathways using the 476 differentially expressed transcripts (fold change ±1.5 and corrected p <0.05) from table S2 that differ between groups. The top section of table shows results for all 476 differentially expressed transcripts, the middle panel shows analysis of only those transcripts that were expressed at higher levels in Sod2^-/-^ cells, while the bottom panel shows only those transcripts that were expressed at lower levels in Sod2^-/-^ cells. In evaluating the significance of identified pathways, a Z score greater than 2 is considered significant. However, the low number of identified genes in the set of transcripts with increased expression in Sod2^-/-^ cells reduces confidence in some assignments. The strongest assignments are to metabolic pathways, splicing, and DNA repair. Several (strong) assignments are based upon overlapping gene sets (predominantly components of the oxidative phosphorylation pathway)—for instance Parkinson's and Huntington's Diseases, where a mitochondrial link to pathogenesis has been identified.
(DOC)

Table S3 **KEGG Pathway Analysis of Entire Microarray Dataset.** As in table S3 above, GeneSifter was used to identify affected KEGG pathways utilizing the entire microarray dataset (>45,000 ‘genes’ on the Affymetrix mouse genome 430 2.0 array). This provides a much broader view of altered metabolic, signal transduction and disease processes that share patterns of gene expression change with those seen in our comparison of Sod2^+/+^ versus Sod2^-/-^ erythroblasts.
(DOC)

Table S4 **Taqman Assays Used for qPCR Validation:** Table S4 lists the endogenous control (18S), analyzed genes and the corresponding inventoried transcript-specific assays (Applied Biosystems Inc, Foster City, CA). Assays were selected with the _m1 suffix; they are designed on exon/intron junctions and do not amplify genomic DNA.
(DOC)
