# Supplementary figures and images for: SOD2 Deficient Erythroid Cells Up-Regulate Transferrin Receptor and Down-Regulate Mitochondrial Biogenesis and Metabolism
Source: PLoS One. 2011 Feb 4;6(2):e16894. doi: 10.1371/journal.pone.0016894 (PMC3033911; doi:10.1371/journal.pone.0016894)

**Figure S1**. **Heat Map of Mitochondrial Ribosomal Protein Gene Expression**


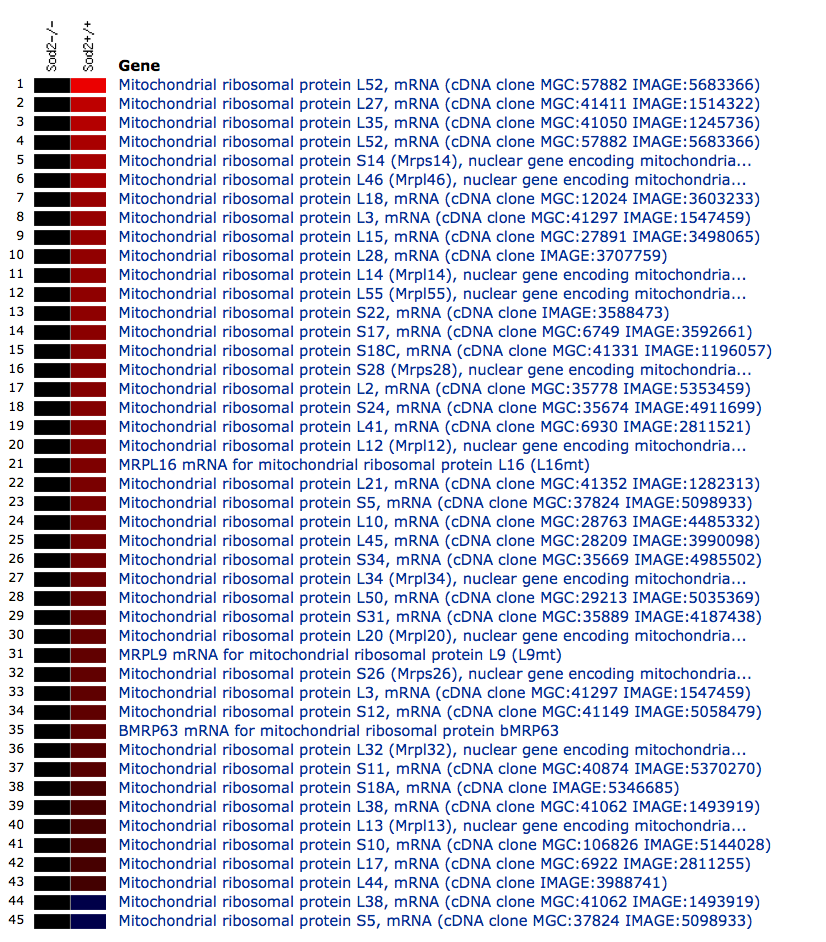

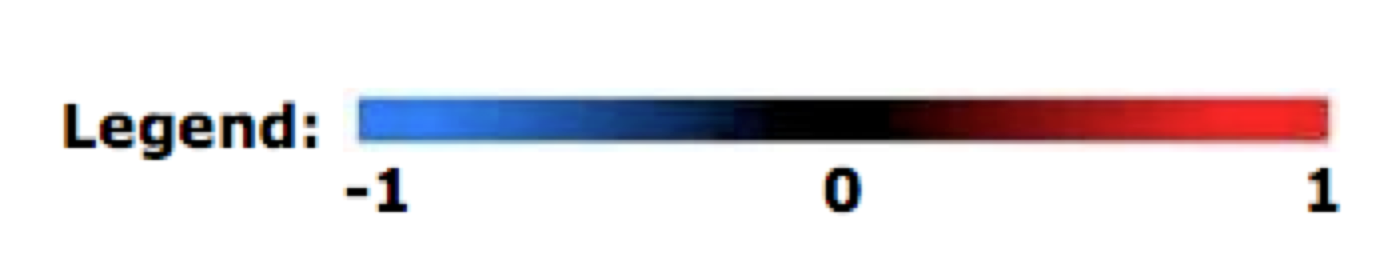

Supplement: Figure S1 — Heat Map of Mitochondrial Ribosomal Protein Gene Expression. 42 Distinct nuclear encoded mitochondrial ribosomal protein genes are expressed differentially when comparing Sod2+/+ and Sod2-/- samples. Data were filtered to display transcripts with ≥1.2 fold change and a corrected p value <0.05. Using these criteria, 43/45 significantly different transcripts are down regulated in Sod2-/- erythroblasts. 3 of the listed genes appear twice and are represented by 2 distinct probe sets. (DOC) [file pone.0016894.s001.doc]
