# Supplementary material for: SOD2 Deficient Erythroid Cells Up-Regulate Transferrin Receptor and Down-Regulate Mitochondrial Biogenesis and Metabolism
Source: PLoS One. 2011 Feb 4;6(2):e16894. doi: 10.1371/journal.pone.0016894 (PMC3033911; doi:10.1371/journal.pone.0016894)
Supplement: Figure S2 — Heat Maps Showing Genes Involved in: Iron Ion Homeostatis, Heme Biosynthesis, or Etiology of Sideroblastic Anemia. Expression of genes annotated as playing a role in iron homeostasis and heme biosynthesis is displayed for both Sod2+/+ and Sod2-/- samples without statistical filtering for fold change or significance. When statistical filters were applied to the set of iron homeostasis genes, ABCb7 and Tfrc were the only genes expressed with a fold change ≥1.5 and p value <0.05. ABCb7 is down in Sod2-/- cells, while Tfrc expression is up. Similarly, genes involved in heme biosynthesis are displayed without filtering on the right. When statistical filters were applied, a putative cDNA (Riken A230051G13) with a proposed role in glycine catabolism and heme biosynthesis was found to be expressed at higher levels in Sod2+/+ cells, meeting the same fold-change and statistical criteria. Finally, genes previously identified as mutated in hereditary sideroblastic anemia were queried for expression. Of these genes, only ABCb7 was (again) found to be significantly differentially expressed. Sod2 appears in the list of iron homeostasis related genes without showing differential expression. This reflects detection of expressed (but deleted for exon 3, and therefore nonfunctional [24]) mRNA in the Sod2-/- cells, as some of the probesets on the microarray for detecting this gene are outside of the deleted exons. (DOC) [file pone.0016894.s002.doc]

**Figure S2**
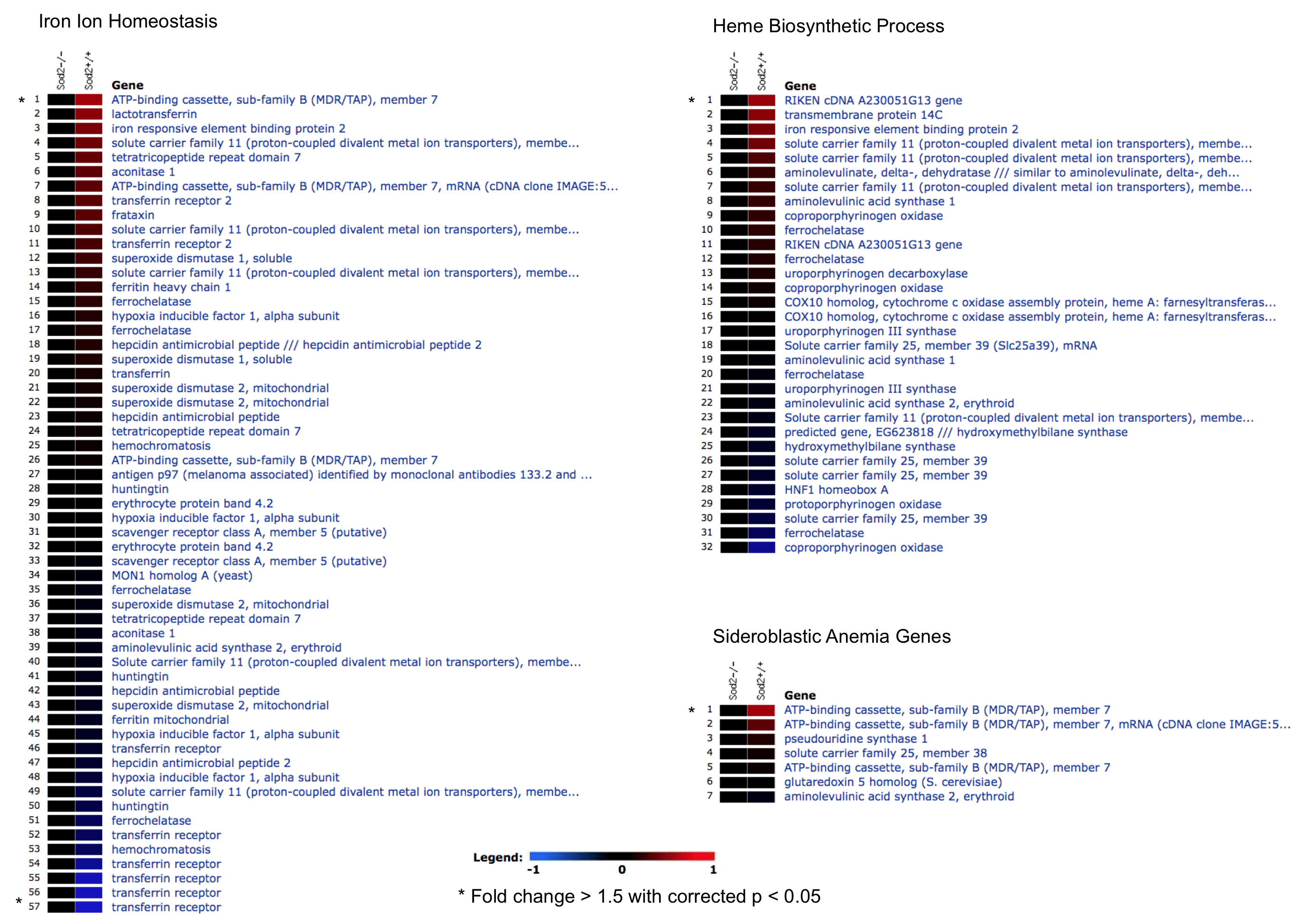
.

**Figure S2.** **Heat Maps Showing Genes Involved in: Iron Ion Homeostatis, Heme Biosynthesis, or Etiology of Sideroblastic Anemia**
