# Supplementary material for: SOD2 Deficient Erythroid Cells Up-Regulate Transferrin Receptor and Down-Regulate Mitochondrial Biogenesis and Metabolism
Source: PLoS One. 2011 Feb 4;6(2):e16894. doi: 10.1371/journal.pone.0016894 (PMC3033911; doi:10.1371/journal.pone.0016894)
Supplement: Table S1 — List of 476 Differentially Expressed Transcripts Comparing Sod2-/- and Sod2+/+ Erythroblast Samples. Criteria used in filtering data were fold change ±1.5 with a corrected p value <0.05 (Benjamini and Hochberg MTC used). (DOC) [file pone.0016894.s003.doc]

**Table S1. List of 476 Differentially Expressed Transcripts Comparing Sod2^-/-^ and Sod2^+/+^ Erythroblast Samples**.

| **Fold Change** | **Direction in Sod2+/+** | | **adj. p-value** | | **Gene Identifier** | | **Gene Name** | **UG Cluster** | **Gene ID** |
| --- | --- | --- | --- | --- | --- | --- | --- | --- | --- |
| 6.24 | Down | | 0.0134 | | NM_008102 | | GTP cyclohydrolase 1, mRNA (cDNA clone MGC:12095 IMAGE:3709238) | Mm.10651 | Gch1 |
| 3.64 | Down | | 0.0170 | | BB698398 | | GTP cyclohydrolase 1, mRNA (cDNA clone MGC:12095 IMAGE:3709238) | Mm.10651 | Gch1 |
| 3.48 | Down | | 0.0241 | | AK010362 | | RIKEN cDNA 2410003J06 gene (2410003J06Rik), transcript variant 1, mRNA | Mm.379207 | 2410003J06Rik |
| 3.18 | Down | | 0.0347 | | M12573 | | Heat shock protein (hsp68) mRNA, clone MHS214 | Mm.372314 | Hspa1b |
| 2.88 | Down | | 0.0399 | | BG076333 | | Methylenetetrahydrofolate dehydrogenase (NAD+ dependent), methenyltetrahydrofolate cyclohydrolase, mRNA (cDNA clone MGC:28481 | Mm.443 | Mthfd2 |
| 2.85 | Down | | 0.0134 | | BB450769 | | ESTs | - | - |
| 2.75 | Down | | 0.0347 | | BG076333 | | Methylenetetrahydrofolate dehydrogenase (NAD+ dependent), methenyltetrahydrofolate cyclohydrolase, mRNA (cDNA clone MGC:28481 | Mm.443 | Mthfd2 |
| 2.75 | Down | | 0.0341 | | M12573 | | Heat shock protein (hsp68) mRNA, clone MHS214 | Mm.372314 | Hspa1b |
| 2.73 | Down | | 0.0090 | | NM_007893 | | Transcription factor phi AP3 | Mm.163132 | E4f1 |
| 2.68 | Down | | 0.0048 | | BB027397 | | Transcribed locus, strongly similar to NP_031919.2 E4F transcription factor 1 [Mus musculus] | Mm.445985 | - |
| 2.66 | Down | | 0.0129 | | BM201499 | | Transcribed locus | Mm.450309 | - |
| 2.59 | Down | | 0.0136 | | BM247240 | | ESTs | - | - |
| 2.36 | Down | | 0.0217 | | BM211430 | | Transcribed locus | Mm.413311 | - |
| 2.35 | Down | | 0.0170 | | BB631473 | | Transcribed locus | Mm.437948 | - |
| 2.33 | Down | | 0.0422 | | NM_013738 | | Pleckstrin 2, mRNA (cDNA clone MGC:18708 IMAGE:4208341) | Mm.103380 | Plek2 |
| 2.31 | Down | | 0.0170 | | BB462549 | | Erythrocyte protein band 4.1, mRNA (cDNA clone IMAGE:3661100) | Mm.30038 | Epb4.1 |
| 2.27 | Down | | 0.0129 | | BB201490 | | Transcribed locus | Mm.445692 | - |
| 2.16 | Down | | 0.0218 | | C79489 | | DNA segment, Chr 5, ERATO Doi 255, expressed | - | - |
| 2.16 | Down | | 0.0391 | | BE631223 | | Transcribed locus | Mm.472907 | - |
| 2.15 | Down | | 0.0240 | | BG066654 | | DNA segment, Chr 10, ERATO Doi 276, expressed | - | - |
| 2.13 | Down | | 0.0182 | | BF461324 | | Transcribed locus | Mm.449557 | - |
| 2.12 | Down | | 0.0129 | | BB205419 | | ESTs | - | - |
| 2.09 | Down | | 0.0324 | | BG071170 | | Ubiquitin specific peptidase 32 (Usp32), mRNA | Mm.178524 | Usp32 |
| 2.05 | Down | | 0.0331 | | NM_009824 | | Core-binding factor, runt domain, alpha subunit 2, translocated to, 3 (human) (Cbfa2t3), transcript variant 1, mRNA | Mm.194339 | Cbfa2t3 |
| 2.04 | Down | | 0.0428 | | BB471309 | | Transcribed locus | Mm.406286 | - |
| 2.03 | Down | | 0.0347 | | AK020483 | | Metastasis associated lung adenocarcinoma transcript 1 (non-coding RNA), mRNA (cDNA clone IMAGE:3582796) | Mm.298256 | Malat1 |
| 2.03 | Down | | 0.0397 | | BM240223 | | Transcribed locus | Mm.438431 | - |
| 2.02 | Down | | 0.0170 | | BB328803 | | Chloride channel 3 (Clcn3), transcript variant a, mRNA | Mm.259751 | Clcn3 |
| 2.01 | Down | | 0.0399 | | AF022992 | | Period homolog 1 (Drosophila), mRNA (cDNA clone MGC:49546 IMAGE:4163009) | Mm.7373 | Per1 |
| 2 | Down | | 0.0397 | | BB547420 | | RIKEN cDNA D930015E06 gene, mRNA (cDNA clone IMAGE:4947647) | Mm.28838 | D930015E06Rik |
| 1.96 | Down | | 0.0324 | | BM225255 | | predicted gene, 100039204 /// predicted gene, 100040620 /// predicted gene, 100040790 /// predicted gene, 100041416 /// predicted gene, 100042151 /// predicted gene, 100042613 /// predicted gene, 100043211 /// predicted gene, 100043316 /// CWC22 spli | - | - |
| 1.95 | Down | | 0.0495 | | BB200185 | | RIKEN cDNA F730015K02 gene | - | - |
| 1.94 | Down | | 0.0287 | | BB047737 | | Transcribed locus | Mm.440651 | - |
| 1.94 | Down | | 0.0350 | | BM225255 | | predicted gene, 100039204 /// predicted gene, 100040620 /// predicted gene, 100040790 /// predicted gene, 100041416 /// predicted gene, 100042151 /// predicted gene, 100043211 /// predicted gene, 100043316 /// CWC22 spliceosome-associated protein hom | - | - |
| 1.94 | Down | | 0.0240 | | AW545973 | | Transcribed locus | Mm.442048 | - |
| 1.92 | Down | | 0.0470 | | BM237980 | | Transcribed locus | Mm.462234 | - |
| 1.91 | Down | | 0.0347 | | BB154631 | | ESTs | - | - |
| 1.9 | Down | | 0.0312 | | BB503322 | | Transcribed locus | Mm.441107 | - |
| 1.9 | Down | | 0.0421 | | AW557531 | | ESTs | - | - |
| 1.89 | Down | | 0.0323 | | BG078867 | | Gametogenetin binding protein 2 (Ggnbp2), mRNA | Mm.356653 | Ggnbp2 |
| 1.88 | Down | | 0.0129 | | BB125494 | | ESTs | - | - |
| 1.88 | Down | | 0.0312 | | AW537781 | | Kinesin family member 24 (Kif24), mRNA | Mm.370288 | Kif24 |
| 1.88 | Down | | 0.0218 | | NM_010023 | | Dodecenoyl-Coenzyme A delta isomerase (3,2 trans-enoyl-Coenyme A isomerase), mRNA (cDNA clone MGC:31498 IMAGE:4487371) | Mm.291743 | Dci |
| 1.87 | Down | | 0.0134 | | AV272467 | | Membrane-associated ring finger (C3HC4) 3, mRNA (cDNA clone MGC:130168 IMAGE:40052074) | Mm.312623 | C3HC4 |
| 1.86 | Down | | 0.0385 | | BB111383 | | Transcribed locus | Mm.416361 | - |
| 1.85 | Down | | 0.0402 | | AI391218 | | Transcribed locus | Mm.471710 | - |
| 1.84 | Down | | 0.0347 | | NM_010265 | | Glucosaminyl (N-acetyl) transferase 1, core 2, mRNA (cDNA clone MGC:11452 IMAGE:3154671) | Mm.244825 | Gcnt1 |
| 1.84 | Down | | 0.0179 | | AK009735 | | adaptor protein complex AP-2, alpha 2 subunit | - | - |
| 1.83 | Down | | 0.0129 | | AA409371 | | RIKEN cDNA 4833412E19 gene | - | - |
| 1.82 | Down | | 0.0331 | | AW551720 | | ESTs | - | - |
| 1.81 | Down | | 0.0331 | | U25708 | | 4F2 heavy chain | Mm.4114 | Slc3a2 |
| 1.8 | Down | | 0.0332 | | BM239430 | | A kinase (PRKA) anchor protein 13 (Akap13), mRNA | Mm.216107 | Akap13 |
| 1.8 | Down | | 0.0447 | | AV271736 | | Spermatogenesis associated 13, mRNA (cDNA clone MGC:170080 IMAGE:8861475) | Mm.149776 | Spata13 |
| 1.79 | Down | | 0.0391 | | AK020830 | | Family with sequence similarity 53, member B, mRNA (cDNA clone MGC:40647 IMAGE:5400742) | Mm.225649 | Fam53b |
| 1.79 | Down | | 0.0343 | | BB009092 | | ESTs | - | - |
| 1.78 | Down | | 0.0250 | | BG069224 | | DNA segment, Chr 10, ERATO Doi 709, expressed | - | - |
| 1.76 | Down | | 0.0397 | | BB502175 | | ESTs | - | - |
| 1.76 | Down | | 0.0422 | | AK013880 | | Asparaginyl-tRNA synthetase (Nars), transcript variant 1, mRNA | Mm.476771 | Nars |
| 1.76 | Down | | 0.0170 | | BG075643 | | ESTs | - | - |
| 1.76 | Down | | 0.0343 | | BM222179 | | ESTs | - | - |
| 1.76 | Down | | 0.0129 | | NM_019581 | | GTP binding protein 2 (Gtpbp2), transcript variant 1, mRNA | Mm.22147 | Gtpbp2 |
| 1.76 | Down | | 0.0419 | | BM293801 | | Transcribed locus | Mm.454419 | - |
| 1.76 | Down | | 0.0090 | | AI606195 | | Erythrocyte protein band 4.1, mRNA (cDNA clone IMAGE:3661100) | Mm.30038 | Epb4.1 |
| 1.75 | Down | | 0.0315 | | BG075096 | | Transcribed locus | Mm.455233 | - |
| 1.74 | Down | | 0.0134 | | BG063165 | | DNA segment, Chr 15, Wayne State University 126, expressed | - | - |
| 1.73 | Down | | 0.0440 | | BB133363 | | ESTs | - | - |
| 1.73 | Down | | 0.0287 | | BB531351 | | Transcribed locus | Mm.440461 | - |
| 1.73 | Down | | 0.0218 | | BB208315 | | Protein kinase for splicing component (Pksc) | Mm.260516 | Crkrs |
| 1.72 | Down | | 0.0134 | | BB391874 | | RIKEN cDNA A630033E08 gene (A630033E08Rik), transcript variant 1, mRNA | Mm.328828 | A630033E08Rik |
| 1.72 | Down | | 0.0021 | | BB029606 | | DCN1, defective in cullin neddylation 1, domain containing 1 (S. cerevisiae), mRNA (cDNA clone MGC:35881 IMAGE:4012394) | Mm.379305 | Dcun1d1 |
| 1.72 | Down | | 0.0447 | | BI465596 | | Transcribed locus | Mm.437701 | - |
| 1.71 | Down | | 0.0129 | | AI849247 | | GTP binding protein 2 (Gtpbp2), transcript variant 1, mRNA | Mm.22147 | Gtpbp2 |
| 1.7 | Down | | 0.0399 | | BB214299 | | Transcribed locus, strongly similar to NP_705766.2 zinc finger, CCHC domain containing 6 [Mus musculus] | Mm.233082 | - |
| 1.69 | Down | | 0.0179 | | AV332053 | | Ankyrin 1, erythroid (Ank1), transcript variant 2, mRNA | Mm.334444 | Ank1 |
| 1.68 | Down | | 0.0347 | | BB550311 | | Transcribed locus | Mm.453379 | - |
| 1.68 | Down | | 0.0331 | | BB136975 | | expressed sequence BB166591 | - | - |
| 1.68 | Down | | 0.0402 | | NM_011538 | | T-box 6 (Tbx6), mRNA | Mm.727 | Tbx6 |
| 1.68 | Down | | 0.0370 | | BF462770 | | Ras-related GTP binding D, mRNA (cDNA clone IMAGE:1349177) | Mm.300814 | Rragd |
| 1.68 | Down | | 0.0240 | | BM224404 | | Transcribed locus, strongly similar to NP_620137.1 FCH domain only 2 [Homo sapiens] | Mm.413221 | - |
| 1.68 | Down | | 0.0170 | | BB555401 | | Transcribed locus | Mm.396090 | - |
| 1.67 | Down | | 0.0343 | | C77595 | | DNA segment, Chr 8, ERATO Doi 51, expressed | - | - |
| 1.67 | Down | | 0.0090 | | AV333182 | | Annexin A7, mRNA (cDNA clone MGC:6143 IMAGE:3157926) | Mm.280231 | Anxa7 |
| 1.67 | Down | | 0.0218 | | BB533969 | | erythrocyte protein band 4.1 | - | - |
| 1.66 | Down | | 0.0186 | | BM210131 | | Transcribed locus, strongly similar to XP_573529.1 PREDICTED: similar to WD-repeat protein 26 [Rattus norvegicus] | Mm.441222 | - |
| 1.66 | Down | | 0.0390 | | BC008997 | | Annexin A7, mRNA (cDNA clone MGC:6143 IMAGE:3157926) | Mm.280231 | Anxa7 |
| 1.66 | Down | | 0.0343 | | AK011813 | | Solute carrier family 16 (monocarboxylic acid transporters), member 10 (Slc16a10), transcript variant 1, mRNA | Mm.186778 | Slc16a10 |
| 1.66 | Down | | 0.0428 | | AI451001 | | Transcribed locus | Mm.448498 | - |
| 1.65 | Down | | 0.0391 | | BB127876 | | Topoisomerase (DNA) I (Top1), mRNA | Mm.217233 | Top1 |
| 1.65 | Down | | 0.0428 | | BB127931 | | RIKEN cDNA 9630026M06 gene | - | - |
| 1.65 | Down | | 0.0127 | | BB163458 | | ESTs | - | - |
| 1.65 | Down | | 0.0240 | | BG918834 | | Meningioma expressed antigen 5 (hyaluronidase) (Mgea5), mRNA | Mm.478002 | Mgea5 |
| 1.64 | Down | | 0.0462 | | BB183534 | | Transcribed locus | Mm.443623 | - |
| 1.64 | Down | | 0.0241 | | BB067736 | | RIKEN cDNA A130086G11 gene | - | - |
| 1.63 | Down | | 0.0315 | | BB153954 | | ESTs | - | - |
| 1.63 | Down | | 0.0287 | | BI076717 | | ESTs | - | - |
| 1.63 | Down | | 0.0170 | | BM245920 | | ESTs, Moderately similar to S12207 hypothetical protein (M.musculus) | - | - |
| 1.63 | Down | | 0.0170 | | BB249892 | | Transcribed locus | Mm.470374 | - |
| 1.62 | Down | | 0.0455 | | BM239301 | | RIKEN cDNA B930068K11 gene | - | - |
| 1.62 | Down | | 0.0134 | | BM243903 | | ESTs | - | - |
| 1.62 | Down | | 0.0316 | | BG070099 | | Transcribed locus | Mm.458987 | - |
| 1.62 | Down | | 0.0391 | | BB251000 | | Transcribed locus | Mm.443495 | - |
| 1.61 | Down | | 0.0347 | | AK012967 | | Kelch-like 25 (Drosophila), mRNA (cDNA clone IMAGE:4159269) | Mm.206223 | Klhl25 |
| 1.61 | Down | | 0.0240 | | BM122196 | | ESTs | - | - |
| 1.6 | Down | | 0.0391 | | BB749838 | | Topoisomerase (DNA) II alpha (Top2a), mRNA | Mm.4237 | Top2a |
| 1.6 | Down | | 0.0315 | | BG073023 | | Transcribed locus | Mm.440673 | - |
| 1.6 | Down | | 0.0129 | | BM231698 | | Transcribed locus | Mm.436906 | - |
| 1.59 | Down | | 0.0333 | | BB041915 | | Transcribed locus | Mm.453339 | - |
| 1.59 | Down | | 0.0312 | | BB222968 | | Transcribed locus | Mm.404218 | - |
| 1.59 | Down | | 0.0370 | | NM_023799 | | Meningioma expressed antigen 5 (hyaluronidase) (Mgea5), mRNA | Mm.478002 | Mgea5 |
| 1.59 | Down | | 0.0192 | | NM_019581 | | GTP binding protein 2 (Gtpbp2), transcript variant 1, mRNA | Mm.22147 | Gtpbp2 |
| 1.59 | Down | | 0.0447 | | BM218282 | | RNA binding motif protein 4 (Rbm4), mRNA | Mm.426069 | Rbm4 |
| 1.59 | Down | | 0.0480 | | BB011550 | | A kinase (PRKA) anchor protein 13 | - | - |
| 1.58 | Down | | 0.0462 | | BC021434 | | Nuclear distribution gene E-like homolog 1 (A. nidulans), mRNA (cDNA clone MGC:29336 IMAGE:5029832) | Mm.31979 | Ndel1 |
| 1.58 | Down | | 0.0421 | | BB076850 | | Transcribed locus | Mm.446212 | - |
| 1.58 | Down | | 0.0420 | | BM212035 | | Transcribed locus | Mm.393076 | - |
| 1.58 | Down | | 0.0265 | | L25109 | | Platelet-activating factor acetylhydrolase, isoform 1b, beta1 subunit, mRNA (cDNA clone MGC:13913 IMAGE:4017963) | Mm.397111 | Pafah1b1 |
| 1.58 | Down | | 0.0371 | | BM242785 | | Eukaryotic translation initiation factor 5, mRNA (cDNA clone MGC:36374 IMAGE:4984912) | Mm.271222 | Eif5 |
| 1.58 | Down | | 0.0315 | | BB736539 | | FH2 domain containing 1, mRNA (cDNA clone MGC:169256 IMAGE:8860651) | Mm.331107 | Fhdc1 |
| 1.57 | Down | | 0.0493 | | BB232083 | | ESTs, Moderately similar to S12207 hypothetical protein (M.musculus) | - | - |
| 1.57 | Down | | 0.0331 | | C79115 | | Transcribed locus | Mm.410441 | - |
| 1.57 | Down | | 0.0283 | | AK017419 | | Transcribed locus | Mm.392103 | - |
| 1.56 | Down | | 0.0408 | | U60330 | | Proteaseome (prosome, macropain) 28 subunit, 3, mRNA (cDNA clone MGC:18383 IMAGE:4221225) | Mm.288477 | Psme3 |
| 1.56 | Down | | 0.0324 | | BB556313 | | Transcribed locus | Mm.411262 | - |
| 1.56 | Down | | 0.0218 | | AI447886 | | Peroxisomal biogenesis factor 12 (Pex12), mRNA | Mm.102205 | Pex12 |
| 1.56 | Down | | 0.0370 | | NM_008748 | | Dual specificity phosphatase 8 (Dusp8), mRNA | Mm.39725 | Dusp8 |
| 1.56 | Down | | 0.0428 | | BB538816 | | Transcribed locus | Mm.440701 | - |
| 1.56 | Down | | 0.0445 | | BG072462 | | Growth arrest and DNA-damage-inducible, gamma interacting protein 1, mRNA (cDNA clone IMAGE:4038217) | - | - |
| 1.56 | Down | | 0.0196 | | AV269574 | | folliculin | - | - |
| 1.55 | Down | | 0.0343 | | AK014775 | | eukaryotic translation initiation factor 2 alpha kinase 1 | - | - |
| 1.55 | Down | | 0.0312 | | BB453509 | | Transcribed locus | Mm.416301 | - |
| 1.55 | Down | | 0.0391 | | BG070640 | | ESTs | - | - |
| 1.55 | Down | | 0.0422 | | BB307279 | | Transcribed locus | Mm.452696 | - |
| 1.55 | Down | | 0.0415 | | BB622498 | | Transcribed locus | Mm.440925 | - |
| 1.55 | Down | | 0.0331 | | AW012617 | | Metastasis associated lung adenocarcinoma transcript 1 (non-coding RNA), mRNA (cDNA clone IMAGE:3582796) | Mm.298256 | Malat1 |
| 1.53 | Down | | 0.0129 | | NM_013785 | | Inositol hexaphosphate kinase 1, mRNA (cDNA clone IMAGE:4483068) | Mm.276155 | Ip6k1 |
| 1.53 | Down | | 0.0312 | | AK014472 | | synuclein, alpha | - | Snca |
| 1.53 | Down | | 0.0170 | | U21209 | | CDC-like kinase 1 (Clk1), transcript variant 2, mRNA | Mm.1761 | Clk1 |
| 1.52 | Down | | 0.0315 | | AW555091 | | ESTs | - | - |
| 1.52 | Down | | 0.0351 | | C78040 | | ESTs, Moderately similar to 60S RIBOSOMAL PROTEIN L34 (R.norvegicus) | - | - |
| 1.52 | Down | | 0.0402 | | AB012273 | | CCAAT/enhancer binding protein (C/EBP), gamma, mRNA (cDNA clone MGC:11669 IMAGE:3709074) | Mm.273090 | Cebpg |
| 1.52 | Down | | 0.0170 | | BB701578 | | FERM domain containing 4A, mRNA (cDNA clone MGC:76363 IMAGE:6830456) | Mm.37932 | Frmd4a |
| 1.52 | Down | | 0.0287 | | BB709476 | | Ring finger protein 10, mRNA (cDNA clone MGC:12014 IMAGE:3602969) | Mm.30051 | Rnf10 |
| 1.52 | Down | | 0.0430 | | BB281000 | | CDNA clone IMAGE:40111838 | Mm.391176 | Cpeb3 |
| 1.51 | Down | | 0.0270 | | BB390252 | | Myotubularin related protein 3 (Mtmr3), mRNA | Mm.425669 | Mtmr3 |
| 1.51 | Down | | 0.0390 | | BB071501 | | RIKEN cDNA 8030497I03 gene | - | - |
| 1.51 | Down | | 0.0240 | | BB206180 | | Transcribed locus | Mm.426397 | - |
| 1.51 | Down | | 0.0287 | | BG067578 | | Guanine nucleotide exchange factor (Larg) | Mm.275266 | Arhgef12 |
| 1.5 | Down | | 0.0351 | | NM_138606 | | Proviral integration site 2 (Pim2), mRNA | Mm.347478 | Pim2 |
| 1.5 | Down | | 0.0218 | | BG074656 | | ESTs | - | - |
|  |  | |  | |  | |  |  |  |
| 20.11 | Up | | 0.0008 | | NM_134126 | | Intraflagellar transport 140 homolog (Chlamydomonas) (Ift140), mRNA | Mm.32802 | Ift140 |
| 4.46 | Up | | 0.0493 | | NM_021301 | | Solute carrier family 15 (H+/peptide transporter), member 2, mRNA (cDNA clone IMAGE:3583898) | Mm.281804 | Slc15a2 |
| 3.96 | Up | | 0.0397 | | AW493533 | | Transcribed locus | Mm.441111 | - |
| 3.54 | Up | | 0.0129 | | AK004898 | | Unkempt-like (Drosophila), mRNA (cDNA clone MGC:183753 IMAGE:9087753) | Mm.267353 | Unkl |
| 3.23 | Up | | 0.0297 | | NM_011921 | | Aldehyde dehydrogenase family 1, subfamily A7, mRNA (cDNA clone IMAGE:1246870) | Mm.14609 | Aldh1a7 |
| 3.04 | Up | | 0.0379 | | BG297038 | | murine leukemia retrovirus | - | - |
| 2.71 | Up | | 0.0420 | | BB251922 | | 2,3-cyclic nucleotide 3 phosphodiesterase | - | - |
| 2.7 | Up | | 0.0347 | | NM_007655 | | CD79A antigen (immunoglobulin-associated alpha), mRNA (cDNA clone MGC:41188 IMAGE:1332396) | Mm.1355 | Cd79a |
| 2.55 | Up | | 0.0440 | | NM_009870 | | Cyclin-dependent kinase 4 (Cdk4), mRNA | Mm.6839 | Cdk4 |
| 2.54 | Up | | 0.0391 | | BM244106 | | Ets transcription factor Spi-B | Mm.8012 | Spib |
| 2.4 | Up | | 0.0411 | | NM_019414 | | Selenium binding protein 2, mRNA (cDNA clone MGC:35927 IMAGE:5096831) | Mm.225405 | Selenbp2 |
| 2.38 | Up | | 0.0240 | | NM_009870 | | Cyclin-dependent kinase 4 (Cdk4), mRNA | Mm.6839 | Cdk4 |
| 2.37 | Up | | 0.0241 | | NM_009127 | | Strain BALB/c stearoyl-coenzyme A desaturase 1 (Scd1) | Mm.267377 | Scd1 |
| 2.33 | Up | | 0.0493 | | NM_008339 | | CD79B antigen, mRNA (cDNA clone MGC:18588 IMAGE:4164551) | Mm.2987 | Cd79b |
| 2.26 | Up | | 0.0170 | | NM_011579 | | T-cell specific GTPase, mRNA (cDNA clone MGC:18429 IMAGE:3662733) | Mm.15793 | Tgtp |
| 2.25 | Up | | 0.0428 | | BB534670 | | Transcribed locus | Mm.406799 | - |
| 2.13 | Up | | 0.0170 | | M58045 | | 2,3-cyclic nucleotide 3 phosphodiesterase, mRNA (cDNA clone MGC:18578 IMAGE:3669076) | Mm.15711 | Cnp |
| 2.13 | Up | | 0.0170 | | NM_008251 | | High mobility group nucleosomal binding domain 1 (Hmgn1), mRNA | Mm.2756 | Hmgn1 |
| 2.1 | Up | | 0.0347 | | U42190 | | MutS homolog 6 (E. coli) (Msh6), mRNA | Mm.18210 | Msh6 |
| 2.08 | Up | | 0.0396 | | BC011223 | | Carbonic anhydrase 1, mRNA (cDNA clone MGC:18584 IMAGE:4208180) | Mm.273195 | Car1 |
| 2.07 | Up | | 0.0315 | | NM_008331 | | Interferon-induced protein with tetratricopeptide repeats 1, mRNA (cDNA clone MGC:5959 IMAGE:3496673) | Mm.439751 | Ifit1 |
| 2.07 | Up | | 0.0428 | | BI144310 | | RIKEN cDNA 2900010J23 gene, mRNA (cDNA clone MGC:8028 IMAGE:3586619) | Mm.27344 | 2900010J23Rik |
| 2.06 | Up | | 0.0493 | | NM_026325 | | Transmembrane protein 179B (Tmem179b), mRNA | Mm.45155 | Tmem179b |
| 2.06 | Up | | 0.0170 | | NM_010708 | | Lectin, galactose binding, soluble 9, mRNA (cDNA clone MGC:5882 IMAGE:3601419) | Mm.341434 | Lgals9 |
| 2.05 | Up | | 0.0347 | | NM_007573 | | Complement component 1, q subcomponent binding protein, mRNA (cDNA clone MGC:46915 IMAGE:3154276) | Mm.30049 | C1qbp |
| 2.05 | Up | | 0.0347 | | NM_026125 | | Family with sequence similarity 132, member A, mRNA (cDNA clone MGC:25716 IMAGE:3966551) | Mm.29140 | Fam132a |
| 2.02 | Up | | 0.0312 | | BB755506 | | Heat shock protein, alpha-crystallin-related, B6 (Hspb6), mRNA | Mm.34885 | Hspb6 |
| 2 | Up | | 0.0241 | | AV116987 | | Hematological and neurological expressed 1-like, mRNA (cDNA clone MGC:91108 IMAGE:30460749) | Mm.371601 | Hn1l |
| 1.98 | Up | | 0.0343 | | BQ176617 | | EF hand domain family A1, mRNA (cDNA clone MGC:37536 IMAGE:4986725) | Mm.26834 | Efha1 |
| 1.97 | Up | | 0.0392 | | NM_011801 | | Craniofacial development protein 1, mRNA (cDNA clone MGC:11431 IMAGE:3964401) | Mm.279437 | Cfdp1 |
| 1.96 | Up | | 0.0250 | | NM_009870 | | Cyclin-dependent kinase 4 (Cdk4), mRNA | Mm.6839 | Cdk4 |
| 1.95 | Up | | 0.0447 | | NM_020558 | | C1D nuclear receptor co-repressor, mRNA (cDNA clone MGC:5983 IMAGE:3591682) | Mm.287982 | C1d |
| 1.95 | Up | | 0.0240 | | NM_134122 | | Nurim (nuclear envelope membrane protein), mRNA (cDNA clone MGC:36566 IMAGE:4984796) | Mm.390992 | Nrm |
| 1.94 | Up | | 0.0315 | | NM_009844 | | CD19 antigen (Cd19), mRNA | Mm.4360 | Cd19 |
| 1.94 | Up | | 0.0170 | | BB759096 | | Tuberous sclerosis 2 (Tsc2), transcript variant 1, mRNA | Mm.30435 | Tsc2 |
| 1.93 | Up | | 0.0343 | | BG792484 | | RIKEN cDNA 1810030N24 gene, mRNA (cDNA clone MGC:41047 IMAGE:1494969) | Mm.28071 | 1810030N24Rik |
| 1.92 | Up | | 0.0428 | | BG073014 | | Single-stranded DNA binding protein 1, mRNA (cDNA clone MGC:41439 IMAGE:1314987) | Mm.276356 | Ssbp1 |
| 1.92 | Up | | 0.0217 | | AK007743 | | Protein disulfide isomerase associated 2 (Pdia2), mRNA | Mm.32631 | Pdia2 |
| 1.92 | Up | | 0.0358 | | BB836564 | | Multiple inositol polyphosphate histidine phosphatase 1, mRNA (cDNA clone MGC:29059 IMAGE:5039704) | Mm.255116 | Minpp1 |
| 1.91 | Up | | 0.0347 | | AU015266 | | Transcribed locus | Mm.466977 | - |
| 1.89 | Up | | 0.0445 | | AV021593 | | Mitochondrial ribosomal protein L52, mRNA (cDNA clone MGC:57882 IMAGE:5683366) | Mm.248163 | Mrpl52 |
| 1.89 | Up | | 0.0312 | | BB279185 | | Progestin and adipoQ receptor family member IV, mRNA (cDNA clone MGC:25089 IMAGE:4500871) | Mm.477803 | Paqr4 |
| 1.88 | Up | | 0.0129 | | NM_007991 | | Fibrillarin, mRNA (cDNA clone MGC:6139 IMAGE:3487295) | Mm.4595 | Fbl |
| 1.87 | Up | | 0.0343 | | BB014781 | | Protein tyrosine phosphatase-like (proline instead of catalytic arginine), member a (Ptpla), transcript variant 1, mRNA | Mm.241205 | Ptpla |
| 1.87 | Up | | 0.0312 | | AF022957 | | Acidic (leucine-rich) nuclear phosphoprotein 32 family, member A (Anp32a), mRNA | Mm.269088 | Anp32a |
| 1.86 | Up | | 0.0235 | | BB199787 | | Transcribed locus | Mm.471437 | - |
| 1.86 | Up | | 0.0402 | | AF220142 | | tripartite motif-containing 34 | - | - |
| 1.86 | Up | | 0.0396 | | NM_019795 | | DnaJ (Hsp40) homolog, subfamily C, member 7, mRNA (cDNA clone MGC:35912 IMAGE:5343384) | Mm.402409 | Dnajc7 |
| 1.85 | Up | | 0.0129 | | NM_026418 | | Regulator of G-protein signalling 10 (Rgs10), mRNA | Mm.18635 | Rgs10 |
| 1.85 | Up | | 0.0165 | | BC026795 | | Replication factor C (activator 1) 3, mRNA (cDNA clone MGC:25594 IMAGE:4015134) | Mm.12553 | Rfc3 |
| 1.85 | Up | | 0.0391 | | NM_010760 | | Mago-nashi homolog, proliferation-associated (Drosophila), mRNA (cDNA clone MGC:5808 IMAGE:3587774) | Mm.808 | Magoh |
| 1.84 | Up | | 0.0331 | | BC011218 | | Elastase 1, pancreatic, mRNA (cDNA clone MGC:19111 IMAGE:4208442) | Mm.2131 | Ela1 |
| 1.84 | Up | | 0.0358 | | AW554405 | | Interferon, alpha-inducible protein 27 like 1, mRNA (cDNA clone MGC:149996 IMAGE:40091489) | Mm.2121 | Ifi27l1 |
| 1.84 | Up | | 0.0295 | | BG072418 | | Ribonuclease III (Rn3) | Mm.293142 | Rnasen |
| 1.83 | Up | | 0.0440 | | BC003830 | | H1 histone family, member 0, mRNA (cDNA clone MGC:19309 IMAGE:4166167) | Mm.24350 | H1f0 |
| 1.83 | Up | | 0.0249 | | AK012340 | | NmrA-like family domain containing 1 (Nmral1), mRNA | Mm.372705 | Nmral1 |
| 1.83 | Up | | 0.0350 | | NM_008894 | | Polymerase (DNA directed), delta 2, regulatory subunit, mRNA (cDNA clone MGC:5849 IMAGE:3257610) | Mm.35788 | Pold2 |
| 1.83 | Up | | 0.0396 | | BB724741 | | calponin 3, acidic /// similar to calponin 3, acidic | - | - |
| 1.82 | Up | | 0.0352 | | AK004595 | | Cytidine monophosphate (UMP-CMP) kinase 2, mitochondrial (Cmpk2), nuclear gene encoding mitochondrial protein, mRNA | Mm.271839 | Cmpk2 |
| 1.82 | Up | | 0.0420 | | NM_139063 | | Muted (Muted), mRNA | Mm.261554 | Muted |
| 1.81 | Up | | 0.0170 | | BM118654 | | PREDICTED: Mus musculus RIKEN cDNA 4930432O21 gene (4930432O21Rik), mRNA | Mm.26540 | 4930432O21Rik |
| 1.81 | Up | | 0.0447 | | AF302653 | | Aspartate-beta-hydroxylase (Asph), transcript variant 2, mRNA | Mm.222206 | Asph |
| 1.81 | Up | | 0.0350 | | BB469322 | | RRM RNA binding protein NSAP1 (Nsap1) | Mm.260545 | Syncrip |
| 1.81 | Up | | 0.0391 | | BI903760 | | Non-SMC element 1 homolog (S. cerevisiae), mRNA (cDNA clone MGC:25737 IMAGE:3984224) | Mm.4467 | Nsmce1 |
| 1.81 | Up | | 0.0316 | | BB283676 | | Tripartite motif-containing 2 (Trim2), mRNA | Mm.44876 | Trim2 |
| 1.8 | Up | | 0.0343 | | AW540162 | | Ring finger protein 20, mRNA (cDNA clone IMAGE:3587961) | Mm.24765 | Rnf20 |
| 1.8 | Up | | 0.0347 | | NM_023422 | | Histone cluster 1, H2bc, mRNA (cDNA clone MGC:30336 IMAGE:3993954) | Mm.261676 | Hist1h2bc |
| 1.79 | Up | | 0.0347 | | AV292968 | | histone deacetylase 2 | - | - |
| 1.79 | Up | | 0.0217 | | AK008404 | | Sorcin (Sri), transcript variant 2, mRNA | Mm.96211 | Sri |
| 1.79 | Up | | 0.0428 | | NM_013663 | | Splicing factor, arginine/serine-rich 3 (SRp20) (Sfrs3), mRNA | Mm.6787 | Sfrs3 |
| 1.79 | Up | | 0.0347 | | NM_010448 | | CArG-binding factor A mRNA, partial cds, alternatively spliced | Mm.280842 | Hnrnpab |
| 1.78 | Up | | 0.0460 | | BQ174458 | | KH-type splicing regulatory protein, mRNA (cDNA clone IMAGE:6848894) | Mm.34296 | Khsrp |
| 1.78 | Up | | 0.0391 | | BC014731 | | Poly (ADP-ribose) polymerase family, member 16, mRNA (cDNA clone IMAGE:4486878) | Mm.31129 | Parp16 |
| 1.78 | Up | | 0.0440 | | AW208944 | | Proteasome (prosome, macropain) 26S subunit, ATPase, 6, mRNA (cDNA clone IMAGE:5351949) | Mm.18472 | Psmc6 |
| 1.78 | Up | | 0.0218 | | BC003335 | | Replication factor C (activator 1) 4 (Rfc4), mRNA | Mm.386835 | Rfc4 |
| 1.77 | Up | | 0.0420 | | AF091101 | | Deoxyuridine triphosphatase, mRNA (cDNA clone MGC:28500 IMAGE:4187559) | Mm.282499 | Dut |
| 1.77 | Up | | 0.0420 | | AW107484 | | Transcribed locus | Mm.393454 | - |
| 1.77 | Up | | 0.0428 | | BM119387 | | Ezrin (Ezr), mRNA | Mm.277812 | Ezr |
| 1.77 | Up | | 0.0391 | | AA409562 | | PRP19/PSO4 pre-mRNA processing factor 19 homolog (S. cerevisiae) (Prpf19), mRNA | Mm.358657 | Prpf19 |
| 1.76 | Up | | 0.0398 | | NM_025424 | | Neuron derived neurotrophic factor (Nenf), mRNA | Mm.46444 | Nenf |
| 1.76 | Up | | 0.0315 | | BC011321 | | DEAD (Asp-Glu-Ala-Asp) box polypeptide 27, mRNA (cDNA clone IMAGE:5363914) | Mm.295031 | Ddx27 |
| 1.76 | Up | | 0.0222 | | NM_011694 | | Voltage-dependent anion channel 1 (Vdac1), mRNA | Mm.3555 | Vdac1 |
| 1.75 | Up | | 0.0397 | | BB278418 | | Cyclin M2 (Cnnm2), transcript variant 1, mRNA | Mm.306903 | Cnnm2 |
| 1.75 | Up | | 0.0397 | | AV339366 | | multiple inositol polyphosphate histidine phosphatase 1 | - | - |
| 1.75 | Up | | 0.0414 | | AK011367 | | Basic transcription factor 3-like 4, mRNA (cDNA clone IMAGE:3495294) | Mm.379178 | Btf3l4 |
| 1.75 | Up | | 0.0420 | | AB067534 | | 2-5 oligoadenylate synthetase 3 (Oas3), mRNA | Mm.204887 | Oas3 |
| 1.74 | Up | | 0.0333 | | BC016273 | | Histidine triad protein member 5 | Mm.229110 | Dcps |
| 1.74 | Up | | 0.0323 | | BB138434 | | scavenger receptor class B, member 1 | - | - |
| 1.74 | Up | | 0.0495 | | BF124540 | | Cysteine and glycine-rich protein 1, mRNA (cDNA clone MGC:6534 IMAGE:2654089) | Mm.196484 | Csrp1 |
| 1.74 | Up | | 0.0414 | | BC004070 | | PRP19/PSO4 pre-mRNA processing factor 19 homolog (S. cerevisiae) (Prpf19), mRNA | Mm.358657 | Prpf19 |
| 1.73 | Up | | 0.0397 | | NM_026632 | | Replication protein A3, mRNA (cDNA clone MGC:41008 IMAGE:1448430) | Mm.29073 | Rpa3 |
| 1.73 | Up | | 0.0347 | | NM_009226 | | Small nuclear ribonucleoprotein D1, mRNA (cDNA clone MGC:18517 IMAGE:4009014) | Mm.603 | Snrpd1 |
| 1.73 | Up | | 0.0445 | | BB529913 | | BTB and CNC homology 2 (Bach2), transcript variant 2, mRNA | Mm.431426 | Bach2 |
| 1.72 | Up | | 0.0350 | | BF682223 | | UDP-glucose ceramide glucosyltransferase (Ugcg), mRNA | Mm.198803 | Ugcg |
| 1.72 | Up | | 0.0347 | | NM_133684 | | MOCO sulphurase C-terminal domain containing 2 (Mosc2), mRNA | Mm.177724 | Mosc2 |
| 1.72 | Up | | 0.0273 | | AK003990 | | Sorting and assembly machinery component 50 homolog (S. cerevisiae), mRNA (cDNA clone MGC:155496 IMAGE:8733929) | Mm.290725 | Samm50 |
| 1.72 | Up | | 0.0343 | | NM_026506 | | Small nuclear ribonucleoprotein polypeptide G, mRNA (cDNA clone MGC:41014 IMAGE:1430048) | Mm.276802 | Snrpg |
| 1.72 | Up | | 0.0422 | | BB611004 | | Transcribed locus, strongly similar to NP_659542.3 tripartite motif-containing 24 [Mus musculus] | Mm.474937 | - |
| 1.72 | Up | | 0.0445 | | NM_016772 | | Enoyl coenzyme A hydratase 1, peroxisomal (Ech1), mRNA | Mm.291776 | Ech1 |
| 1.72 | Up | | 0.0358 | | NM_019673 | | Actin-like 6A, mRNA (cDNA clone MGC:5731 IMAGE:3491205) | Mm.41077 | Actl6a |
| 1.71 | Up | | 0.0391 | | BB795103 | | Transcribed locus | Mm.393454 | - |
| 1.71 | Up | | 0.0331 | | AF204156 | | LSM2 homolog, U6 small nuclear RNA associated (S. cerevisiae), mRNA (cDNA clone MGC:13889 IMAGE:3982410) | Mm.165735 | Lsm2 |
| 1.71 | Up | | 0.0393 | | BF319868 | | Protein disulfide isomerase associated 3, mRNA (cDNA clone MGC:28333 IMAGE:4016310) | Mm.263177 | Pdia3 |
| 1.71 | Up | | 0.0399 | | BF228243 | | Leptin receptor overlapping transcript, mRNA (cDNA clone MGC:11607 IMAGE:2651399) | Mm.4756 | Leprot |
| 1.71 | Up | | 0.0331 | | BG066664 | | Fermitin family homolog 3 (Drosophila) (Fermt3), mRNA | Mm.157591 | Fermt3 |
| 1.71 | Up | | 0.0480 | | NM_026506 | | Small nuclear ribonucleoprotein polypeptide G, mRNA (cDNA clone MGC:41014 IMAGE:1430048) | Mm.276802 | Snrpg |
| 1.71 | Up | | 0.0308 | | BM120662 | | Cleavage stimulation factor, 3 pre-RNA subunit 2, mRNA (cDNA clone MGC:36412 IMAGE:5322335) | Mm.67938 | Cstf2 |
| 1.7 | Up | | 0.0323 | | AK004632 | | Golgi associated, gamma adaptin ear containing, ARF binding protein 2 (Gga2), mRNA | Mm.29619 | Gga2 |
| 1.7 | Up | | 0.0312 | | AV296905 | | Metastasis associated protein MTA2 (MTA2) | Mm.25339 | Mta2 |
| 1.7 | Up | | 0.0421 | | AA756810 | | STT3, subunit of the oligosaccharyltransferase complex, homolog A (S. cerevisiae), mRNA (cDNA clone MGC:46919 IMAGE:5353797) | Mm.2863 | Stt3a |
| 1.69 | Up | | 0.0399 | | BB183081 | | Glial fibrillary acidic protein [Mus musculus], mRNA sequence | Mm.1239 | Gfap |
| 1.69 | Up | | 0.0391 | | BC020080 | | Transmembrane protein 140, mRNA (cDNA clone MGC:38515 IMAGE:5352975) | Mm.248440 | Tmem140 |
| 1.69 | Up | | 0.0389 | | AK006541 | | Acyl-CoA synthetase long-chain family member 5, mRNA (cDNA clone MGC:18968 IMAGE:3987201) | Mm.292056 | Acsl5 |
| 1.69 | Up | | 0.0365 | | BC005704 | | Lysine-rich coiled-coil 1 (Krcc1), mRNA | Mm.250569 | Krcc1 |
| 1.69 | Up | | 0.0439 | | BB311524 | | Zinc finger protein 39, mRNA (cDNA clone IMAGE:4016059) | Mm.127646 | Zfp39 |
| 1.69 | Up | | 0.0424 | | BC009141 | | Splicing factor 3a, subunit 3 (Sf3a3), mRNA | Mm.25779 | Sf3a3 |
| 1.69 | Up | | 0.0396 | | BM239527 | | Cleavage and polyadenylation specific factor 2, mRNA (cDNA clone MGC:5855 IMAGE:3497854) | Mm.716 | Cpsf2 |
| 1.69 | Up | | 0.0129 | | NM_026312 | | RIKEN cDNA 2610029G23 gene (2610029G23Rik), mRNA | Mm.273405 | 2610029G23Rik |
| 1.69 | Up | | 0.0281 | | BB807707 | | Tetraspanin 13, mRNA (cDNA clone MGC:18777 IMAGE:4166625) | Mm.254663 | Tspan13 |
| 1.68 | Up | | 0.0447 | | NM_019484 | | RNA and export factor binding protein 2 (Refbp2), mRNA | Mm.389208 | Refbp2 |
| 1.68 | Up | | 0.0312 | | NM_025573 | | Splicing factor, arginine/serine rich 9, mRNA (cDNA clone MGC:7233 IMAGE:3483731) | Mm.287826 | Sfrs9 |
| 1.68 | Up | | 0.0327 | | NM_053161 | | Mitochondrial ribosomal protein L27, mRNA (cDNA clone MGC:41411 IMAGE:1514322) | Mm.34951 | Mrpl27 |
| 1.68 | Up | | 0.0396 | | AK017673 | | RIKEN cDNA 2810417H13 gene, mRNA (cDNA clone MGC:29306 IMAGE:5006272) | Mm.351273 | 2810417H13Rik |
| 1.68 | Up | | 0.0391 | | AV328340 | | Eukaryotic translation initiation factor 2-alpha kinase 2, mRNA (cDNA clone MGC:11397 IMAGE:3964935) | Mm.378990 | Eif2ak2 |
| 1.68 | Up | | 0.0347 | | AV225029 | | Transmembrane emp24 protein transport domain containing 7, mRNA (cDNA clone IMAGE:3985067) | Mm.296043 | Tmed7 |
| 1.68 | Up | | 0.0493 | | NM_021793 | | Transmembrane protein 8 (five membrane-spanning domains), mRNA (cDNA clone MGC:11794 IMAGE:3595205) | Mm.304656 | Tmem8 |
| 1.67 | Up | | 0.0170 | | BC024400 | | Coiled-coil domain containing 5 (Ccdc5), mRNA | Mm.39293 | Ccdc5 |
| 1.67 | Up | | 0.0347 | | NM_025319 | | RIKEN cDNA 0610009B22 gene, mRNA (cDNA clone MGC:35774 IMAGE:5009365) | Mm.35693 | 0610009B22Rik |
| 1.67 | Up | | 0.0455 | | BC003914 | | 3-hydroxyisobutyrate dehydrogenase (Hibadh), mRNA | Mm.286458 | Hibadh |
| 1.66 | Up | | 0.0445 | | NM_133655 | | CD81 antigen (Cd81), mRNA | Mm.806 | Cd81 |
| 1.66 | Up | | 0.0478 | | BF580235 | | Mouse endogenous murine mink cell focus-forming (MCF) envelope protein mRNA, 3 end, clone T-7.2 | - | - |
| 1.66 | Up | | 0.0428 | | NM_029571 | | KTI12 homolog, chromatin associated (S. cerevisiae) (Kti12), mRNA | Mm.440007 | Kti12 |
| 1.66 | Up | | 0.0290 | | NM_133744 | | Coiled-coil domain containing 71 (Ccdc71), mRNA | Mm.478066 | Ccdc71 |
| 1.66 | Up | | 0.0414 | | NM_029397 | | Swan mRNA, complete cds; alternatively spliced | Mm.27660 | Rbm12 |
| 1.66 | Up | | 0.0473 | | BG070487 | | Acetyl-CoA acetyltransferase (Acat1 gene) | Mm.293233 | Acat1 |
| 1.66 | Up | | 0.0499 | | BC012260 | | Proteasome (prosome, macropain) inhibitor subunit 1, mRNA (cDNA clone IMAGE:40109778) | Mm.146984 | Psmf1 |
| 1.65 | Up | | 0.0305 | | BC004739 | | Ring finger protein 26 (Rnf26), mRNA | Mm.472711 | Rnf26 |
| 1.65 | Up | | 0.0343 | | NM_133676 | | O-sialoglycoprotein endopeptidase (Osgep), mRNA | Mm.274791 | Osgep |
| 1.65 | Up | | 0.0170 | | BC005598 | | Eukaryotic translation initiation factor 3, subunit M (Eif3m), mRNA | Mm.379278 | Eif3m |
| 1.65 | Up | | 0.0461 | | NM_026086 | | N-acetylneuraminic acid phosphatase, mRNA (cDNA clone MGC:27982 IMAGE:3596532) | Mm.120911 | 4930519N13Rik |
| 1.65 | Up | | 0.0315 | | BC024693 | | Prefoldin 1, mRNA (cDNA clone MGC:19010 IMAGE:4020579) | Mm.30184 | Pfdn1 |
| 1.64 | Up | | 0.0350 | | NM_007531 | | Prohibitin 2 (Phb2), mRNA | Mm.36241 | Phb2 |
| 1.64 | Up | | 0.0347 | | AK014889 | | Death associated protein 3, mRNA (cDNA clone MGC:28659 IMAGE:4235517) | Mm.29028 | Dap3 |
| 1.64 | Up | | 0.0312 | | BC013545 | | Centrin 2, mRNA (cDNA clone MGC:19232 IMAGE:4242430) | Mm.24643 | Cetn2 |
| 1.64 | Up | | 0.0324 | | NM_010247 | | X-ray repair complementing defective repair in Chinese hamster cells 6, mRNA (cDNA clone MGC:18310 IMAGE:3669715) | Mm.288809 | Xrcc6 |
| 1.64 | Up | | 0.0337 | | NM_024220 | | NADH dehydrogenase (ubiquinone) 1, subcomplex unknown, 2 (Ndufc2), mRNA | Mm.334031 | Ndufc2 |
| 1.64 | Up | | 0.0362 | | NM_008229 | | Histone deacetylase 2 (Hdac2), mRNA | Mm.19806 | Hdac2 |
| 1.64 | Up | | 0.0312 | | NM_011189 | | Proteasome (prosome, macropain) 28 subunit, alpha (Psme1), mRNA | Mm.830 | Psme1 |
| 1.64 | Up | | 0.0290 | | NM_026635 | | Family with sequence similarity 96, member A, mRNA (cDNA clone MGC:11942 IMAGE:3600028) | Mm.27107 | Fam96a |
| 1.64 | Up | | 0.0391 | | BC027370 | | Centlein, centrosomal protein, mRNA (cDNA clone IMAGE:4948503) | Mm.440562 | Cntln |
| 1.64 | Up | | 0.0470 | | BC002240 | | Dpy-30 homolog (C. elegans) (Dpy30), mRNA | Mm.28536 | Dpy30 |
| 1.64 | Up | | 0.0399 | | BB747462 | | Transmembrane 9 superfamily member 2 (Tm9sf2), mRNA | Mm.275191 | Tm9sf2 |
| 1.64 | Up | | 0.0241 | | NM_138745 | | Methylenetetrahydrofolate dehydrogenase (NADP+ dependent), methenyltetrahydrofolate cyclohydrolase, formyltetrahydrofolate sy | Mm.29584 | Mthfd1 |
| 1.63 | Up | | 0.0346 | | BF787384 | | Mitochondrial ribosomal protein L35, mRNA (cDNA clone MGC:41050 IMAGE:1245736) | Mm.28579 | Mrpl35 |
| 1.63 | Up | | 0.0331 | | AK014330 | | Histone aminotransferase 1, mRNA (cDNA clone IMAGE:5025987) | Mm.272472 | Hat1 |
| 1.63 | Up | | 0.0170 | | AK005498 | | nucleophosmin 1 | - | - |
| 1.63 | Up | | 0.0457 | | BM227771 | | Transcribed locus | Mm.458563 | - |
| 1.63 | Up | | 0.0470 | | NM_008188 | | THUMP domain containing 3, mRNA (cDNA clone MGC:13936 IMAGE:3987438) | Mm.781 | Thumpd3 |
| 1.63 | Up | | 0.0297 | | BG073415 | | Transcribed locus | Mm.405067 | - |
| 1.63 | Up | | 0.0445 | | BG070689 | | Nudix (nucleoside diphosphate linked moiety X)-type motif 19 (Nudt19), mRNA | Mm.410206 | Nudt19 |
| 1.62 | Up | | 0.0397 | | NM_134138 | | Proteasome (prosome, macropain) assembly chaperone 2 (Psmg2), mRNA | Mm.150701 | Psmg2 |
| 1.62 | Up | | 0.0343 | | NM_019685 | | RuvB-like protein 1, mRNA (cDNA clone MGC:5718 IMAGE:3582426) | Mm.42195 | Ruvbl1 |
| 1.62 | Up | | 0.0218 | | BC005632 | | Family with sequence similarity 108, member A (Fam108a), mRNA | Mm.29802 | Fam108a |
| 1.62 | Up | | 0.0321 | | NM_025380 | | Eukaryotic translation elongation factor 1 epsilon 1 (Eef1e1), mRNA | Mm.477755 | Eef1e1 |
| 1.61 | Up | | 0.0232 | | BB071632 | | Enhancer of rudimentary homolog (Drosophila) (Erh), mRNA | Mm.378913 | Erh |
| 1.61 | Up | | 0.0218 | | BI413749 | | Family with sequence similarity 108, member A (Fam108a), mRNA | Mm.29802 | Fam108a |
| 1.61 | Up | | 0.0420 | | BG065061 | | Methionine adenosyltransferase II, alpha (Mat2a), mRNA | Mm.29815 | Mat2a |
| 1.61 | Up | | 0.0445 | | NM_019708 | | Short coiled-coil protein, mRNA (cDNA clone IMAGE:4506964) | Mm.246911 | Scoc |
| 1.61 | Up | | 0.0180 | | BI901126 | | Growth factor, erv1 (S. cerevisiae)-like (augmenter of liver regeneration), mRNA (cDNA clone MGC:35826 IMAGE:5342526) | Mm.28124 | Gfer |
| 1.61 | Up | | 0.0142 | | NM_011150 | | Lectin, galactoside-binding, soluble, 3 binding protein (Lgals3bp), mRNA | Mm.3152 | Lgals3bp |
| 1.61 | Up | | 0.0312 | | NM_016763 | | Hydroxysteroid (17-beta) dehydrogenase 10, mRNA (cDNA clone MGC:41111 IMAGE:3376098) | Mm.6994 | Hsd17b10 |
| 1.61 | Up | | 0.0397 | | NM_026000 | | Proteasome (prosome, macropain) 26S subunit, non-ATPase, 9 (Psmd9), mRNA | Mm.278997 | Psmd9 |
| 1.61 | Up | | 0.0315 | | AF068749 | | Sphingosine kinase 1, mRNA (cDNA clone MGC:47288 IMAGE:4221357) | Mm.20944 | Sphk1 |
| 1.61 | Up | | 0.0445 | | BF322051 | | Anaphase promoting complex subunit 10, mRNA (cDNA clone MGC:25547 IMAGE:3710036) | Mm.257445 | Anapc10 |
| 1.61 | Up | | 0.0250 | | AV297071 | | Novel nuclear protein Nnp1 variant 1 | Mm.38344 | Rrp1 |
| 1.61 | Up | | 0.0445 | | NM_025520 | | LSM5 homolog, U6 small nuclear RNA associated (S. cerevisiae) (Lsm5), mRNA | Mm.25642 | Lsm5 |
| 1.61 | Up | | 0.0440 | | NM_008613 | | Meiosis-specific nuclear structural protein 1 (Mns1), mRNA | Mm.387671 | Mns1 |
| 1.6 | Up | | 0.0392 | | NM_015751 | | ATP-binding cassette, sub-family E (OABP), member 1, mRNA (cDNA clone MGC:5781 IMAGE:3489529) | Mm.5831 | Abce1 |
| 1.6 | Up | | 0.0320 | | NM_013929 | | SIVA1, apoptosis-inducing factor, mRNA (cDNA clone MGC:18759 IMAGE:4013026) | Mm.289812 | Siva1 |
| 1.6 | Up | | 0.0479 | | BB783243 | | RIKEN cDNA 2310005N03 gene (2310005N03Rik), mRNA | Mm.86589 | 2310005N03Rik |
| 1.6 | Up | | 0.0421 | | BC013777 | | Small nuclear ribonucleoprotein polypeptide A, mRNA (cDNA clone MGC:25531 IMAGE:3588138) | Mm.413078 | Snrpa1 |
| 1.6 | Up | | 0.0343 | | BC004578 | | Replication protein A2, mRNA (cDNA clone MGC:6146 IMAGE:3586727) | Mm.2870 | Rpa2 |
| 1.6 | Up | | 0.0365 | | NM_030695 | | LPS-responsive beige-like anchor (Lrba), transcript variant 1, mRNA | Mm.439825 | Lrba |
| 1.6 | Up | | 0.0459 | | AK015136 | | CDNA clone IMAGE:5012232 | Mm.116284 | 4932415G12Rik |
| 1.6 | Up | | 0.0428 | | NM_007530 | | B-cell receptor-associated protein 29, mRNA (cDNA clone MGC:14077 IMAGE:2646883) | Mm.242379 | Bcap29 |
| 1.6 | Up | | 0.0326 | | AY034062 | | Splicing factor proline/glutamine rich (polypyrimidine tract binding protein associated) (Sfpq), mRNA | Mm.257276 | Sfpq |
| 1.6 | Up | | 0.0343 | | NM_009156 | | Selenoprotein W | Mm.42829 | Sepw1 |
| 1.59 | Up | | 0.0392 | | NM_134024 | | Tubulin, gamma 1 (Tubg1), mRNA | Mm.142348 | Tubg1 |
| 1.59 | Up | | 0.0222 | | NM_027141 | | SPRY domain-containing SOCS box protein SSB-3 | Mm.274794 | Spsb3 |
| 1.59 | Up | | 0.0250 | | BB315728 | | Nuclear factor I/X, mRNA (cDNA clone MGC:5944 IMAGE:3491917) | Mm.9394 | Nfix |
| 1.59 | Up | | 0.0499 | | BQ174458 | | KH-type splicing regulatory protein, mRNA (cDNA clone IMAGE:6848894) | Mm.34296 | Khsrp |
| 1.59 | Up | | 0.0283 | | NM_009746 | | B-cell CLL/lymphoma 7C, mRNA (cDNA clone MGC:11464 IMAGE:3153237) | Mm.89667 | Bcl7c |
| 1.59 | Up | | 0.0436 | | BB523428 | | RIKEN cDNA D930014E17 gene (D930014E17Rik), mRNA | Mm.334138 | D930014E17Rik |
| 1.59 | Up | | 0.0391 | | U48737 | | PRP4 pre-mRNA processing factor 4 homolog B (yeast) (Prpf4b), mRNA | Mm.10027 | Prpf4b |
| 1.59 | Up | | 0.0331 | | NM_009514 | | Pre-B lymphocyte gene 3 (Vpreb3), mRNA | Mm.333851 | Vpreb3 |
| 1.59 | Up | | 0.0331 | | AY075132 | | Interferon induced with helicase C domain 1 (Ifih1), mRNA | Mm.136224 | Ifih1 |
| 1.59 | Up | | 0.0241 | | BF228243 | | Leptin receptor overlapping transcript, mRNA (cDNA clone MGC:11607 IMAGE:2651399) | Mm.4756 | Leprot |
| 1.59 | Up | | 0.0365 | | NM_025321 | | Succinate dehydrogenase complex, subunit C, integral membrane protein, mRNA (cDNA clone MGC:6555 IMAGE:2810852) | Mm.198138 | Pcp4l1 |
| 1.59 | Up | | 0.0356 | | BG070487 | | Acetyl-CoA acetyltransferase (Acat1 gene) | Mm.293233 | Acat1 |
| 1.59 | Up | | 0.0343 | | BC006028 | | 5-nucleotidase, cytosolic II | - | - |
| 1.59 | Up | | 0.0414 | | NM_025419 | | RIKEN cDNA 1110059G10 gene, mRNA (cDNA clone MGC:31735 IMAGE:4921846) | Mm.268307 | 1110059G10Rik |
| 1.59 | Up | | 0.0331 | | AY007382 | | Phospholipase A2, group XIIA, mRNA (cDNA clone MGC:25458 IMAGE:4456431) | Mm.151951 | Pla2g12a |
| 1.58 | Up | | 0.0365 | | BI655075 | | Interferon, alpha-inducible protein 27 like 1, mRNA (cDNA clone MGC:149996 IMAGE:40091489) | Mm.2121 | Ifi27l1 |
| 1.58 | Up | | 0.0273 | | NM_008330 | | Interferon gamma inducible protein 47, mRNA (cDNA clone MGC:11403 IMAGE:2651113) | Mm.24769 | Olfr56 |
| 1.58 | Up | | 0.0323 | | NM_010256 | | Phosphoribosylglycinamide formyltransferase (Gart), mRNA | Mm.4505 | Gart |
| 1.58 | Up | | 0.0343 | | BC010826 | | Family with sequence similarity 162, member A (Fam162a), mRNA | Mm.212991 | Fam162a |
| 1.58 | Up | | 0.0459 | | BI905689 | | NADH dehydrogenase (ubiquinone) 1 beta subcomplex, 10, mRNA (cDNA clone MGC:36216 IMAGE:4219237) | Mm.1129 | Ndufb10 |
| 1.58 | Up | | 0.0240 | | AV223474 | | zinc finger, DHHC domain containing 14 | - | - |
| 1.58 | Up | | 0.0350 | | BE370775 | | zinc finger, CCHC domain containing 11 | - | - |
| 1.58 | Up | | 0.0312 | | BC006809 | | Phosphomannomutase 1, mRNA (cDNA clone MGC:11610 IMAGE:3153981) | Mm.18939 | Pmm1 |
| 1.58 | Up | | 0.0493 | | NM_020022 | | Replication factor C (activator 1) 2, mRNA (cDNA clone MGC:35852 IMAGE:5374851) | Mm.332739 | Rfc2 |
| 1.58 | Up | | 0.0493 | | NM_030082 | | Histone cluster 3, H2ba (Hist3h2ba), mRNA | Mm.28022 | Hist3h2ba |
| 1.58 | Up | | 0.0415 | | AK013131 | | Pentatricopeptide repeat domain 3 (Ptcd3), mRNA | Mm.30256 | Ptcd3 |
| 1.58 | Up | | 0.0328 | | NM_011956 | | Nucleotide binding protein 2, mRNA (cDNA clone MGC:13715 IMAGE:4038123) | Mm.36718 | Nubp2 |
| 1.57 | Up | | 0.0397 | | BI525140 | | Plectin 1, mRNA (cDNA clone IMAGE:5097449) | Mm.373750 | Plec1 |
| 1.57 | Up | | 0.0392 | | BB818311 | | Transcribed locus | Mm.435639 | - |
| 1.57 | Up | | 0.0493 | | NM_009547 | | Zinc finger protein 161 (Zfp161), mRNA | Mm.29434 | Zfp161 |
| 1.57 | Up | | 0.0440 | | NM_023431 | | Melanoma associated antigen (mutated) 1, mRNA (cDNA clone IMAGE:5369109) | Mm.273418 | Mum1 |
| 1.57 | Up | | 0.0455 | | NM_008095 | | Glioblastoma amplified sequence (Gbas), mRNA | Mm.12468 | Gbas |
| 1.57 | Up | | 0.0398 | | BI246587 | | Mitochondrial ribosomal protein S14 (Mrps14), nuclear gene encoding mitochondrial protein, mRNA | Mm.29599 | Mrps14 |
| 1.57 | Up | | 0.0392 | | AK013287 | | N-acetyltransferase 13 (Nat13), mRNA | Mm.278726 | Nat13 |
| 1.57 | Up | | 0.0315 | | AI510077 | | Cullin associated and neddylation disassociated 1 (Cand1), mRNA | Mm.203965 | Cand1 |
| 1.57 | Up | | 0.0343 | | AK011546 | | Mitochondrial poly(A) polymerase (Mtpap), nuclear gene encoding mitochondrial protein, mRNA | Mm.49826 | Mtpap |
| 1.57 | Up | | 0.0475 | | AW060738 | | Nucleolar protein 7 (Nol7), mRNA | Mm.439927 | Nol7 |
| 1.57 | Up | | 0.0218 | | NM_023331 | | Mitochondrial ribosomal protein L46 (Mrpl46), nuclear gene encoding mitochondrial protein, mRNA | Mm.193181 | Mrpl46 |
| 1.56 | Up | | 0.0274 | | NM_029787 | | Cytochrome b5 reductase 3, mRNA (cDNA clone MGC:36220 IMAGE:4238275) | Mm.22560 | Cyb5r3 |
| 1.56 | Up | | 0.0222 | | BC003288 | | DNA segment, Chr 10, Wayne State University 52, expressed (D10Wsu52e), mRNA | Mm.9257 | D10Wsu52e |
| 1.56 | Up | | 0.0424 | | BM119407 | | ATP-binding cassette, sub-family B (MDR/TAP), member 7, mRNA (cDNA clone IMAGE:5368040) | Mm.426128 | Abcb7 |
| 1.56 | Up | | 0.0421 | | BI080799 | | PREDICTED: Mus musculus small nuclear ribonucleoprotein polypeptide F (Snrpf), mRNA | Mm.259642 | Snrpf |
| 1.56 | Up | | 0.0273 | | BC025501 | | Achalasia, adrenocortical insufficiency, alacrimia (Aaas), mRNA | Mm.352946 | Aaas |
| 1.56 | Up | | 0.0347 | | AF176524 | | F-box protein FBL10 | Mm.86406 | Kdm2b |
| 1.56 | Up | | 0.0347 | | AB026997 | | Calpastatin, mRNA (cDNA clone MGC:12116 IMAGE:3710078) | Mm.29163 | Cast |
| 1.56 | Up | | 0.0397 | | BC024945 | | RIKEN cDNA 9430016H08 gene, mRNA (cDNA clone MGC:28199 IMAGE:3989472) | Mm.275428 | 9430016H08Rik |
| 1.55 | Up | | 0.0170 | | NM_013908 | | F-box and WD-40 domain protein 5, mRNA (cDNA clone MGC:18679 IMAGE:4211592) | Mm.29170 | Fbxw5 |
| 1.55 | Up | | 0.0315 | | AK016023 | | Glycerophosphodiester phosphodiesterase domain containing 1 (Gdpd1), mRNA | Mm.477731 | Gdpd1 |
| 1.55 | Up | | 0.0240 | | NM_008801 | | Phosphodiesterase 6D, cGMP-specific, rod, delta, mRNA (cDNA clone MGC:11435 IMAGE:3964336) | Mm.12925 | Pde6d |
| 1.55 | Up | | 0.0408 | | BF158817 | | Transcribed locus, strongly similar to NP_001014139.1 ubiquitin-like domain containing CTD phosphatase 1 [Rattus norvegicus] | Mm.397554 | - |
| 1.55 | Up | | 0.0315 | | NM_025358 | | NADH dehydrogenase (ubiquinone) 1 alpha subcomplex, 9, mRNA (cDNA clone IMAGE:3601317) | Mm.29939 | Ndufa9 |
| 1.55 | Up | | 0.0134 | | AK004129 | | Cytosolic iron-sulfur protein assembly 1 homolog (S. cerevisiae), mRNA (cDNA clone MGC:8301 IMAGE:3593736) | Mm.45179 | Ciao1 |
| 1.55 | Up | | 0.0179 | | BB667651 | | Pyruvate kinase liver and red blood cell (Pklr), nuclear gene encoding mitochondrial protein, transcript variant 1, mRNA | Mm.383180 | Pklr |
| 1.55 | Up | | 0.0347 | | AK020708 | | Glycoprotein, synaptic 2 (Gpsn2), mRNA | Mm.352239 | Gpsn2 |
| 1.55 | Up | | 0.0170 | | NM_010360 | | Glutathione S-transferase, mu 5, mRNA (cDNA clone MGC:11497 IMAGE:3154170) | Mm.282351 | Gstm5 |
| 1.55 | Up | | 0.0392 | | BG064779 | | Expressed sequence AU021838 (AU021838), mRNA | Mm.268180 | AU021838 |
| 1.55 | Up | | 0.0170 | | AA920031 | | Nuclear transport factor 2, mRNA (cDNA clone MGC:7387 IMAGE:3487899) | Mm.269188 | Nutf2 |
| 1.55 | Up | | 0.0428 | | BM210281 | | Transcribed locus, strongly similar to NP_001028856.1 chaperonin containing Tcp1, subunit 6A (zeta 1) [Rattus norvegicus] | Mm.153159 | - |
| 1.55 | Up | | 0.0331 | | AK007572 | | Zinc finger, DHHC domain containing 4 (Zdhhc4), mRNA | Mm.261606 | Zdhhc4 |
| 1.55 | Up | | 0.0355 | | NM_134040 | | DEAD (Asp-Glu-Ala-Asp) box polypeptide 1 (Ddx1), mRNA | Mm.251255 | Ddx1 |
| 1.55 | Up | | 0.0397 | | NM_010874 | | N-acetyltransferase 2 (arylamine N-acetyltransferase), mRNA (cDNA clone MGC:14025 IMAGE:3992093) | Mm.4695 | Nat2 |
| 1.55 | Up | | 0.0379 | | BC002162 | | Centrin 3, mRNA (cDNA clone MGC:7312 IMAGE:3485864) | Mm.12481 | Cetn3 |
| 1.55 | Up | | 0.0396 | | BI078449 | | Signal peptidase complex subunit 2 homolog (S. cerevisiae), mRNA (cDNA clone IMAGE:3582190) | Mm.30043 | Spcs2 |
| 1.55 | Up | | 0.0470 | | BF453953 | | RIKEN cDNA 2610036L11 gene, mRNA (cDNA clone MGC:182866 IMAGE:9087480) | Mm.251779 | 2610036L11Rik |
| 1.55 | Up | | 0.0399 | | BF158817 | | Transcribed locus, strongly similar to NP_001014139.1 ubiquitin-like domain containing CTD phosphatase 1 [Rattus norvegicus] | Mm.397554 | - |
| 1.55 | Up | | 0.0351 | | AV135835 | | Pinin (Pnn), mRNA | Mm.22347 | Pnn |
| 1.54 | Up | | 0.0347 | | U00431 | | High mobility group box 1, mRNA (cDNA clone MGC:8044 IMAGE:3587109) | Mm.313345 | Hmgb1 |
| 1.54 | Up | | 0.0428 | | BC025116 | | Cbp/p300-interacting transactivator, with Glu/Asp-rich carboxy-terminal domain, 4, mRNA (cDNA clone IMAGE:3670674) | Mm.272646 | Cited4 |
| 1.54 | Up | | 0.0343 | | NM_008253 | | High mobility group box 3, mRNA (cDNA clone MGC:18367 IMAGE:3980194) | Mm.336087 | Hmgb3 |
| 1.54 | Up | | 0.0235 | | U31934 | | Ubiquitin-conjugating enzyme | Mm.384234 | Ube2i |
| 1.54 | Up | | 0.0312 | | NM_023136 | | Deoxythymidylate kinase, mRNA (cDNA clone MGC:29227 IMAGE:5039765) | Mm.250332 | Dtymk |
| 1.54 | Up | | 0.0403 | | BG067031 | | NMDA receptor-regulated gene 1, mRNA (cDNA clone IMAGE:3674167) | Mm.275281 | Narg1 |
| 1.54 | Up | | 0.0347 | | AK010292 | | Ribonuclease H2, large subunit, mRNA (cDNA clone MGC:48163 IMAGE:1493985) | Mm.182470 | Rnaseh2a |
| 1.54 | Up | | 0.0240 | | NM_007838 | | Dolichyl-di-phosphooligosaccharide-protein glycotransferase (Ddost), mRNA | Mm.7236 | Ddost |
| 1.54 | Up | | 0.0347 | | AF098949 | | Demethyl-Q 7, mRNA (cDNA clone MGC:48033 IMAGE:1380996) | Mm.20634 | Coq7 |
| 1.54 | Up | | 0.0129 | | AK017830 | | DnaJ (Hsp40) homolog, subfamily C, member 14 (Dnajc14), mRNA | Mm.296915 | Dnajc14 |
| 1.54 | Up | | 0.0347 | | BC011114 | | NADH dehydrogenase (ubiquinone) 1 alpha subcomplex, 4, mRNA (cDNA clone MGC:7218 IMAGE:3483028) | Mm.477723 | Ndufa4 |
| 1.54 | Up | | 0.0424 | | M25487 | | Histone H2b mRNA, 3 end | Mm.264645 | Hist1h2bp |
| 1.54 | Up | | 0.0493 | | AW553435 | | Isoleucine-tRNA synthetase 2, mitochondrial, mRNA (cDNA clone IMAGE:4922885) | Mm.331142 | Iars2 |
| 1.54 | Up | | 0.0250 | | AK019868 | | Polymerase (RNA) III (DNA directed) polypeptide H (Polr3h), mRNA | Mm.215115 | Polr3h |
| 1.54 | Up | 0.0358 | | BI658327 | | Minichromosome maintenance deficient 3 (S. cerevisiae), mRNA (cDNA clone MGC:30531 IMAGE:5007886) | | Mm.4502 | Mcm3 |
| 1.54 | Up | 0.0397 | | NM_026533 | | Ribosomal protein S13 (Rps13), mRNA | | Mm.14798 | Rps13 |
| 1.54 | Up | 0.0381 | | NM_026503 | | RIKEN cDNA 1110058L19 gene, mRNA (cDNA clone MGC:35651 IMAGE:5367409) | | Mm.250424 | 1110058L19Rik |
| 1.54 | Up | 0.0420 | | AF356874 | | Histidine triad protein 3 | | Mm.25285 | Hint2 |
| 1.53 | Up | 0.0483 | | NM_008303 | | Heat shock protein 1 (chaperonin 10), mRNA (cDNA clone MGC:36134 IMAGE:5004427) | | Mm.215667 | Hspe1 |
| 1.53 | Up | 0.0401 | | BB230296 | | COMM domain containing 3 (Commd3), mRNA | | Mm.249586 | Commd3 |
| 1.53 | Up | 0.0445 | | BB729616 | | NOP58 ribonucleoprotein homolog (yeast) (Nop58), mRNA | | Mm.220367 | Nop58 |
| 1.53 | Up | 0.0422 | | NM_018762 | | Glycoprotein 9 (platelet) (Gp9), mRNA | | Mm.12897 | Gp9 |
| 1.53 | Up | 0.0387 | | AA409562 | | PRP19/PSO4 pre-mRNA processing factor 19 homolog (S. cerevisiae) (Prpf19), mRNA | | Mm.358657 | Prpf19 |
| 1.53 | Up | 0.0391 | | BC016676 | | Vaccinia related kinase 1, mRNA (cDNA clone MGC:12003 IMAGE:3602292) | | Mm.2981 | Vrk1 |
| 1.53 | Up | 0.0251 | | M33934 | | Inosine 5-phosphate dehydrogenase 2, mRNA (cDNA clone MGC:6193 IMAGE:2646679) | | Mm.6065 | Impdh2 |
| 1.53 | Up | 0.0493 | | BB429200 | | Transcribed locus | | Mm.470916 | - |
| 1.53 | Up | 0.0312 | | AK013962 | | Succinate dehydrogenase complex, subunit D, integral membrane protein, mRNA (cDNA clone MGC:175620 IMAGE:40131036) | | Mm.10406 | Sdhd |
| 1.53 | Up | 0.0347 | | AK007703 | | ATP-binding cassette transporter ABCA3 | | Mm.477776 | Abca3 |
| 1.53 | Up | 0.0445 | | AW553381 | | RIKEN cDNA C030046I01 gene, mRNA (cDNA clone IMAGE:5029949) | | Mm.300416 | C030046I01Rik |
| 1.53 | Up | 0.0343 | | BI692577 | | NADH dehydrogenase (ubiquinone) flavoprotein 2, mRNA (cDNA clone MGC:32165 IMAGE:5003524) | | Mm.2206 | Ndufv2 |
| 1.53 | Up | 0.0312 | | BB174377 | | RIKEN cDNA A230051G13 gene, mRNA (cDNA clone MGC:107430 IMAGE:6432747) | | Mm.266175 | A230051G13Rik |
| 1.53 | Up | 0.0312 | | NM_007422 | | Adenylosuccinate synthetase, non muscle (Adss), mRNA | | Mm.338021 | Adss |
| 1.52 | Up | 0.0287 | | AF316999 | | DEXH (Asp-Glu-X-His) box polypeptide 58, mRNA (cDNA clone MGC:35613 IMAGE:2651254) | | Mm.271830 | Dhx58 |
| 1.52 | Up | 0.0129 | | AI480598 | | Eukaryotic translation initiation factor 2B, subunit 3 (Eif2b3), transcript variant 1, mRNA | | Mm.34612 | Eif2b3 |
| 1.52 | Up | 0.0170 | | NM_027139 | | TAF9 RNA polymerase II, TATA box binding protein (TBP)-associated factor, mRNA (cDNA clone MGC:28404 IMAGE:4024616) | | Mm.301148 | Taf9 |
| 1.52 | Up | 0.0455 | | AK013709 | | CArG-binding factor A mRNA, partial cds, alternatively spliced | | Mm.280842 | Hnrnpab |
| 1.52 | Up | 0.0428 | | AV158882 | | NAD(P)H dehydrogenase, quinone 1 (Nqo1), mRNA | | Mm.252 | Nqo1 |
| 1.52 | Up | 0.0420 | | AK002873 | | RIKEN cDNA 2810002N01 gene (2810002N01Rik), mRNA | | Mm.425762 | 2810002N01Rik |
| 1.52 | Up | 0.0240 | | BF468377 | | Vacuolar protein sorting 52 (yeast) (Vps52), mRNA | | Mm.296044 | Vps52 |
| 1.52 | Up | 0.0283 | | BC007472 | | Polypyrimidine tract binding protein 1, mRNA (cDNA clone MGC:25792 IMAGE:4022742) | | Mm.265610 | Ptbp1 |
| 1.52 | Up | 0.0479 | | BG919982 | | Transcribed locus | | Mm.474025 | - |
| 1.52 | Up | 0.0204 | | AK003393 | | isocitrate dehydrogenase 3 (NAD+) alpha | | - | - |
| 1.52 | Up | 0.0390 | | NM_019953 | | Canopy 2 homolog (zebrafish), mRNA (cDNA clone MGC:6853 IMAGE:2650612) | | Mm.44220 | Cnpy2 |
| 1.52 | Up | 0.0241 | | BB787147 | | Propionyl Coenzyme A carboxylase, beta polypeptide, mRNA (cDNA clone MGC:46787 IMAGE:5098790) | | Mm.335385 | Pccb |
| 1.52 | Up | 0.0312 | | BB406585 | | Solute carrier family 38, member 10, mRNA (cDNA clone IMAGE:5011722) | | Mm.253403 | Slc38a10 |
| 1.52 | Up | 0.0396 | | NM_009041 | | Radixin, mRNA (cDNA clone IMAGE:3484334) | | Mm.245746 | Rdx |
| 1.52 | Up | 0.0218 | | BB494388 | | Malonyl CoA:ACP acyltransferase (mitochondrial), mRNA (cDNA clone IMAGE:5321973) | | Mm.37560 | Mcat |
| 1.52 | Up | 0.0460 | | BC006960 | | Sorting nexin 2, mRNA (cDNA clone MGC:6322 IMAGE:2812557) | | Mm.252171 | Snx2 |
| 1.52 | Up | 0.0315 | | BC024615 | | RIKEN cDNA 1110008P14 gene, mRNA (cDNA clone MGC:19388 IMAGE:2812475) | | Mm.27608 | 1110008P14Rik |
| 1.52 | Up | 0.0350 | | BG066125 | | Proteasome (prosome, macropain) subunit, alpha type 4, mRNA (cDNA clone MGC:5640 IMAGE:3592024) | | Mm.30270 | Psma4 |
| 1.52 | Up | 0.0343 | | NM_133991 | | FtsJ homolog 1 (E. coli), mRNA (cDNA clone MGC:41313 IMAGE:3468633) | | Mm.327529 | Ftsj1 |
| 1.51 | Up | 0.0378 | | NM_013715 | | Kip1 C-terminus interacting protein-2 (Kic2) | | Mm.402384 | Cops5 |
| 1.51 | Up | 0.0422 | | BI408935 | | Translocase of inner mitochondrial membrane 50 homolog (yeast) (Timm50), nuclear gene encoding mitochondrial protein, mRNA | | Mm.167913 | Timm50 |
| 1.51 | Up | 0.0370 | | AK006658 | | RIKEN cDNA 1700040I03 gene (1700040I03Rik), mRNA | | Mm.379221 | 1700040I03Rik |
| 1.51 | Up | 0.0420 | | NM_026310 | | Mitochondrial ribosomal protein L18, mRNA (cDNA clone MGC:12024 IMAGE:3603233) | | Mm.290166 | Mrpl18 |
| 1.51 | Up | 0.0424 | | BB043703 | | O-sialoglycoprotein endopeptidase-like 1, mRNA (cDNA clone IMAGE:5053559) | | Mm.212846 | Ormdl1 |
| 1.51 | Up | 0.0496 | | BM119274 | | COP9 (constitutive photomorphogenic) homolog, subunit 2 (Arabidopsis thaliana), mRNA (cDNA clone MGC:18349 IMAGE:3675508) | | Mm.3596 | Cops2 |
| 1.51 | Up | 0.0408 | | BM213850 | | Nucleolar and coiled-body phosphoprotein 1 (Nolc1), transcript variant 1, mRNA | | Mm.402190 | Nolc1 |
| 1.51 | Up | 0.0399 | | AB049633 | | Mitochondrial ribosomal protein L3, mRNA (cDNA clone MGC:41297 IMAGE:1547459) | | Mm.29746 | Mrpl3 |
| 1.51 | Up | 0.0428 | | AK005233 | | metaxin 2 | | - | - |
| 1.51 | Up | 0.0241 | | NM_053092 | | Lysyl-tRNA synthetase, mRNA (cDNA clone MGC:6923 IMAGE:2811286) | | Mm.196544 | Kars |
| 1.51 | Up | 0.0129 | | NM_007636 | | Chaperonin containing Tcp1, subunit 2 (beta), mRNA (cDNA clone MGC:25091 IMAGE:4500896) | | Mm.247788 | Cct2 |
| 1.51 | Up | 0.0397 | | NM_133981 | | Asparagine-linked glycosylation 9 homolog (yeast, alpha 1,2 mannosyltransferase) (Alg9), mRNA | | Mm.160035 | Alg9 |
| 1.51 | Up | 0.0312 | | AK005025 | | Rcd1 (required for cell differentiation) homolog 1 (S. pombe), mRNA (cDNA clone IMAGE:5039514) | | Mm.291708 | Rqcd1 |
| 1.51 | Up | 0.0170 | | NM_015765 | | Heat shock protein 14, mRNA (cDNA clone MGC:6095 IMAGE:3493119) | | Mm.89341 | Hspa14 |
| 1.51 | Up | 0.0420 | | NM_019767 | | Actin related protein 2/3 complex, subunit 1A, mRNA (cDNA clone MGC:5680 IMAGE:3485409) | | Mm.371610 | Arpc1a |
| 1.51 | Up | 0.0367 | | BM120802 | | RIKEN cDNA 2010321M09 gene, mRNA (cDNA clone MGC:28004 IMAGE:3602181) | | Mm.272616 | 2010321M09Rik |
| 1.51 | Up | 0.0134 | | BF134272 | | SET translocation, mRNA (cDNA clone MGC:19002 IMAGE:4014298) | | Mm.335942 | Set |
| 1.51 | Up | 0.0445 | | BB497590 | | Succinate dehydrogenase complex, subunit D, integral membrane protein, mRNA (cDNA clone MGC:175620 IMAGE:40131036) | | Mm.10406 | Sdhd |
| 1.5 | Up | 0.0456 | | BC012404 | | Peroxisomal membrane protein 3, mRNA (cDNA clone MGC:11449 IMAGE:3964491) | | Mm.132336 | Pxmp3 |
| 1.5 | Up | 0.0323 | | BC012707 | | Glutathione S-transferase, theta 2, mRNA (cDNA clone MGC:13991 IMAGE:3994154) | | Mm.24118 | Gstt2 |
| 1.5 | Up | 0.0403 | | AK002902 | | Translocase of outer mitochondrial membrane 20 homolog (yeast) (Tomm20), nuclear gene encoding mitochondrial protein, mRNA | | Mm.380026 | Tomm20 |
| 1.5 | Up | 0.0397 | | BE984258 | | Brain and reproductive organ-expressed protein isoform IV (Bre) mRNA, complete cds; alternatively spliced | | Mm.249822 | Bre |
| 1.5 | Up | 0.0405 | | AW556558 | | Ring finger protein 213, mRNA (cDNA clone IMAGE:3966957) | | Mm.133342 | Rnf213 |
