# Supplementary material for: SOD2 Deficient Erythroid Cells Up-Regulate Transferrin Receptor and Down-Regulate Mitochondrial Biogenesis and Metabolism
Source: PLoS One. 2011 Feb 4;6(2):e16894. doi: 10.1371/journal.pone.0016894 (PMC3033911; doi:10.1371/journal.pone.0016894)
Supplement: Table S2 — KEGG Pathway Analysis of the 476 most highly differentially expressed genes. The GeneSifter program was used to generate a list of KEGG pathways using the 476 differentially expressed transcripts (fold change ±1.5 and corrected p <0.05) from table S2 that differ between groups. The top section of table shows results for all 476 differentially expressed transcripts, the middle panel shows analysis of only those transcripts that were expressed at higher levels in Sod2-/- cells, while the bottom panel shows only those transcripts that were expressed at lower levels in Sod2-/- cells. In evaluating the significance of identified pathways, a Z score greater than 2 is considered significant. However, the low number of identified genes in the set of transcripts with increased expression in Sod2-/- cells reduces confidence in some assignments. The strongest assignments are to metabolic pathways, splicing, and DNA repair. Several (strong) assignments are based upon overlapping gene sets (predominantly components of the oxidative phosphorylation pathway)—for instance Parkinson's and Huntington's Diseases, where a mitochondrial link to pathogenesis has been identified. (DOC) [file pone.0016894.s004.doc]

**Table S2. KEGG Pathway Analysis of the 476 most highly differentially expressed genes.**

| **ALL 476 Differential Transcripts** |  |  |  |
| --- | --- | --- | --- |
| **KEGG Pathway** | **List** | **Gene Set** | **z-score** |
| Metabolic pathways | 41 | 1061 | 2.71 |
| Spliceosome | 11 | 118 | 4.52 |
| DNA replication | 9 | 35 | 8.46 |
| Huntington's disease | 9 | 160 | 2.34 |
| Purine metabolism | 9 | 150 | 2.55 |
| Mismatch repair | 8 | 22 | 9.8 |
| Parkinson's disease | 8 | 109 | 3.04 |
| Nucleotide excision repair | 7 | 42 | 5.63 |
| Pyrimidine metabolism | 6 | 92 | 2.3 |
| Peroxisome | 5 | 77 | 2.09 |
| Proteasome | 5 | 44 | 3.58 |
| RNA degradation | 5 | 55 | 2.96 |
| Homologous recombination | 4 | 27 | 3.91 |
| Valine, leucine and isoleucine degradation | 4 | 45 | 2.59 |
| One carbon pool by folate | 3 | 15 | 4.16 |
| Glyoxylate and dicarboxylate metabolism | 2 | 16 | 2.43 |
| Fatty acid biosynthesis | 1 | 6 | 2.12 |
|  |  |  |  |
|  |  |  |  |
| **149 Transcripts Increased in Sod2-/-** |  |  |  |
| **KEGG Pathway** | **List** | **Gene Set** | **z-score** |
| Antigen processing and presentation | 2 | 71 | 3.34 |
| Alanine, aspartate and glutamate metabolism | 1 | 31 | 2.57 |
| Aminoacyl-tRNA biosynthesis | 1 | 42 | 2.1 |
| Circadian rhythm - mammal | 1 | 13 | 4.27 |
| Ether lipid metabolism | 1 | 34 | 2.42 |
| Fatty acid metabolism | 1 | 40 | 2.18 |
| Folate biosynthesis | 1 | 11 | 4.68 |
| Glyoxylate and dicarboxylate metabolism | 1 | 16 | 3.8 |
| Nitrogen metabolism | 1 | 22 | 3.17 |
| O-Glycan biosynthesis | 1 | 23 | 3.08 |
| One carbon pool by folate | 1 | 15 | 3.94 |
| Proteasome | 1 | 44 | 2.04 |
| Vasopressin-regulated water reabsorption | 1 | 42 | 2.1 |
|  |  |  |  |
|  |  |  |  |
| **327 Transcripts Decreased in Sod2-/-** |  |  |  |
| **KEGG Pathway** | **List** | **Gene Set** | **z-score** |
| Metabolic pathways | 36 | 1061 | 2.7 |
| Spliceosome | 10 | 118 | 4.53 |
| DNA replication | 9 | 35 | 9.27 |
| Purine metabolism | 9 | 150 | 3.07 |
| Huntington's disease | 8 | 160 | 2.32 |
| Mismatch repair | 8 | 22 | 10.68 |
| Nucleotide excision repair | 7 | 42 | 6.24 |
| Parkinson's disease | 7 | 109 | 2.9 |
| Oxidative phosphorylation | 6 | 110 | 2.23 |
| Pyrimidine metabolism | 6 | 92 | 2.73 |
| RNA degradation | 5 | 55 | 3.38 |
| Homologous recombination | 4 | 27 | 4.35 |
| Proteasome | 4 | 44 | 3.02 |
| Valine, leucine and isoleucine degradation | 4 | 45 | 2.96 |
| One carbon pool by folate | 2 | 15 | 2.86 |
| Protein export | 2 | 22 | 2.13 |
| Fatty acid biosynthesis | 1 | 6 | 2.35 |
| MAPK signaling pathway | 1 | 263 | -2.14 |
| Ubiquinone and other terpenoid-quinone biosynthesis | 1 | 7 | 2.12 |
