# Supplementary material for: SOD2 Deficient Erythroid Cells Up-Regulate Transferrin Receptor and Down-Regulate Mitochondrial Biogenesis and Metabolism
Source: PLoS One. 2011 Feb 4;6(2):e16894. doi: 10.1371/journal.pone.0016894 (PMC3033911; doi:10.1371/journal.pone.0016894)
Supplement: Table S3 — KEGG Pathway Analysis of Entire Microarray Dataset. As in table S3 above, GeneSifter was used to identify affected KEGG pathways utilizing the entire microarray dataset (>45,000 ‘genes’ on the Affymetrix mouse genome 430 2.0 array). This provides a much broader view of altered metabolic, signal transduction and disease processes that share patterns of gene expression change with those seen in our comparison of Sod2+/+ versus Sod2-/- erythroblasts. (DOC) [file pone.0016894.s005.doc]

**Table S3. KEGG Pathway Analysis of Entire Microarray Dataset.**

| **Entire murine Array (>45,000 features)** |  |  |  |  |  |  |
| --- | --- | --- | --- | --- | --- | --- |
| **KEGG Pathway** | **List** | **Up in Sod2+/+** | **Down in Sod2+/+** | **Gene Set** | **z-score (Up)** | **z-score (Down)** |
| Spliceosome | 118 | 110 | 8 | 118 | 9 | -9 |
| Neuroactive ligand-receptor interaction | 285 | 78 | 207 | 285 | -8.72 | 8.72 |
| Ribosome | 86 | 83 | 3 | 86 | 8.27 | -8.27 |
| Oxidative phosphorylation | 110 | 94 | 16 | 110 | 7.03 | -7.03 |
| Cell cycle | 123 | 101 | 22 | 123 | 6.69 | -6.69 |
| Cytokine-cytokine receptor interaction | 232 | 72 | 160 | 232 | -6.67 | 6.67 |
| Parkinson's disease | 109 | 91 | 18 | 109 | 6.58 | -6.58 |
| Arachidonic acid metabolism | 74 | 14 | 60 | 74 | -5.81 | 5.81 |
| DNA replication | 35 | 35 | 0 | 35 | 5.66 | -5.66 |
| Alzheimer's disease | 154 | 115 | 39 | 154 | 5.64 | -5.64 |
| Huntington's disease | 160 | 118 | 42 | 160 | 5.51 | -5.51 |
| RNA degradation | 55 | 48 | 7 | 55 | 5.21 | -5.21 |
| Calcium signaling pathway | 172 | 58 | 114 | 172 | -4.99 | 4.99 |
| Proteasome | 44 | 39 | 5 | 44 | 4.84 | -4.84 |
| Pyrimidine metabolism | 92 | 71 | 21 | 92 | 4.81 | -4.81 |
| Nucleotide excision repair | 42 | 37 | 5 | 42 | 4.66 | -4.66 |
| Oocyte meiosis | 110 | 81 | 29 | 110 | 4.52 | -4.52 |
| Mismatch repair | 22 | 22 | 0 | 22 | 4.48 | -4.48 |
| Linoleic acid metabolism | 40 | 7 | 33 | 40 | -4.43 | 4.43 |
| Citrate cycle (TCA cycle) | 29 | 26 | 3 | 29 | 4.03 | -4.03 |
| Basal cell carcinoma | 55 | 14 | 41 | 55 | -4.02 | 4.02 |
| RNA polymerase | 25 | 23 | 2 | 25 | 3.98 | -3.98 |
| Purine metabolism | 150 | 102 | 48 | 150 | 3.9 | -3.9 |
| ECM-receptor interaction | 76 | 23 | 53 | 76 | -3.89 | 3.89 |
| Axon guidance | 127 | 45 | 82 | 127 | -3.87 | 3.87 |
| Retinol metabolism | 56 | 15 | 41 | 56 | -3.86 | 3.86 |
| Cell adhesion molecules (CAMs) | 131 | 47 | 84 | 131 | -3.83 | 3.83 |
| Progesterone-mediated oocyte maturation | 86 | 62 | 24 | 86 | 3.7 | -3.7 |
| Olfactory transduction | 64 | 19 | 45 | 64 | -3.66 | 3.66 |
| Propanoate metabolism | 29 | 25 | 4 | 29 | 3.66 | -3.66 |
| Ubiquitin mediated proteolysis | 134 | 91 | 43 | 134 | 3.66 | -3.66 |
| Vascular smooth muscle contraction | 122 | 44 | 78 | 122 | -3.65 | 3.65 |
| B cell receptor signaling pathway | 76 | 55 | 21 | 76 | 3.52 | -3.52 |
| Steroid hormone biosynthesis | 45 | 12 | 33 | 45 | -3.47 | 3.47 |
| Homologous recombination | 27 | 23 | 4 | 27 | 3.42 | -3.42 |
| Hypertrophic cardiomyopathy (HCM) | 85 | 29 | 56 | 85 | -3.4 | 3.4 |
| Metabolic pathways | 1061 | 603 | 458 | 1061 | 3.31 | -3.31 |
| Neurotrophin signaling pathway | 126 | 84 | 42 | 126 | 3.26 | -3.26 |
| Protein export | 22 | 19 | 3 | 22 | 3.2 | -3.2 |
| Glycine, serine and threonine metabolism | 30 | 7 | 23 | 30 | -3.19 | 3.19 |
| Acute myeloid leukemia | 56 | 41 | 15 | 56 | 3.14 | -3.14 |
| MAPK signaling pathway | 263 | 113 | 150 | 263 | -3.14 | 3.14 |
| Complement and coagulation cascades | 68 | 23 | 45 | 68 | -3.08 | 3.08 |
| alpha-Linolenic acid metabolism | 18 | 3 | 15 | 18 | -3.04 | 3.04 |
| Arrhythmogenic right ventricular cardiomyopathy (ARVC) | 72 | 25 | 47 | 72 | -3.02 | 3.02 |
| Chronic myeloid leukemia | 73 | 51 | 22 | 73 | 3.02 | -3.02 |
| Melanogenesis | 99 | 37 | 62 | 99 | -3.02 | 3.02 |
| Non-small cell lung cancer | 54 | 39 | 15 | 54 | 2.94 | -2.94 |
| Basal transcription factors | 32 | 25 | 7 | 32 | 2.93 | -2.93 |
| Leishmaniasis | 66 | 46 | 20 | 66 | 2.84 | -2.84 |
| Metabolism of xenobiotics by cytochrome P450 | 61 | 21 | 40 | 61 | -2.82 | 2.82 |
| SNARE interactions in vesicular transport | 34 | 26 | 8 | 34 | 2.82 | -2.82 |
| Colorectal cancer | 63 | 44 | 19 | 63 | 2.8 | -2.8 |
| Nitrogen metabolism | 22 | 5 | 17 | 22 | -2.79 | 2.79 |
| Pancreatic cancer | 70 | 48 | 22 | 70 | 2.74 | -2.74 |
| Glioma | 64 | 44 | 20 | 64 | 2.64 | -2.64 |
| ABC transporters | 43 | 14 | 29 | 43 | -2.61 | 2.61 |
| Drug metabolism - cytochrome P450 | 68 | 25 | 43 | 68 | -2.59 | 2.59 |
| Base excision repair | 32 | 24 | 8 | 32 | 2.57 | -2.57 |
| Lysosome | 117 | 75 | 42 | 117 | 2.57 | -2.57 |
| Fc gamma R-mediated phagocytosis | 87 | 57 | 30 | 87 | 2.48 | -2.48 |
| Long-term depression | 69 | 26 | 43 | 69 | -2.46 | 2.46 |
| Endometrial cancer | 52 | 36 | 16 | 52 | 2.45 | -2.45 |
| Histidine metabolism | 25 | 7 | 18 | 25 | -2.45 | 2.45 |
| Pyruvate metabolism | 39 | 28 | 11 | 39 | 2.44 | -2.44 |
| Dilated cardiomyopathy | 88 | 35 | 53 | 88 | -2.39 | 2.39 |
| Apoptosis | 85 | 55 | 30 | 85 | 2.3 | -2.3 |
| Malaria | 47 | 17 | 30 | 47 | -2.23 | 2.23 |
| Valine, leucine and isoleucine degradation | 45 | 31 | 14 | 45 | 2.23 | -2.23 |
| p53 signaling pathway | 67 | 44 | 23 | 67 | 2.2 | -2.2 |
| Phenylalanine metabolism | 16 | 4 | 12 | 16 | -2.2 | 2.2 |
| Tyrosine metabolism | 33 | 11 | 22 | 33 | -2.2 | 2.2 |
| Thyroid cancer | 29 | 21 | 8 | 29 | 2.17 | -2.17 |
| Renal cell carcinoma | 69 | 45 | 24 | 69 | 2.15 | -2.15 |
| Jak-STAT signaling pathway | 138 | 60 | 78 | 138 | -2.12 | 2.12 |
| PPAR signaling pathway | 70 | 28 | 42 | 70 | -2.09 | 2.09 |
| Maturity onset diabetes of the young | 25 | 8 | 17 | 25 | -2.04 | 2.04 |
| Arginine and proline metabolism | 52 | 20 | 32 | 52 | -2.02 | 2.02 |
| Ether lipid metabolism | 34 | 12 | 22 | 34 | -2 | 2 |
