# Supplementary material for: SOD2 Deficient Erythroid Cells Up-Regulate Transferrin Receptor and Down-Regulate Mitochondrial Biogenesis and Metabolism
Source: PLoS One. 2011 Feb 4;6(2):e16894. doi: 10.1371/journal.pone.0016894 (PMC3033911; doi:10.1371/journal.pone.0016894)
Supplement: Table S4 — Taqman Assays Used for qPCR Validation: Table S4 lists the endogenous control (18S), analyzed genes and the corresponding inventoried transcript-specific assays (Applied Biosystems Inc, Foster City, CA). Assays were selected with the _m1 suffix; they are designed on exon/intron junctions and do not amplify genomic DNA. (DOC) [file pone.0016894.s006.doc]

**Table S4. Taqman Assays Used for qPCR Validation**

| **Assay ID** | **Gene Symbol** | **Gene Name** | **Amplicon length** |
| --- | --- | --- | --- |
| 4333760F | 18S | Eukaryotic 18S rRNA Endogenous Control | 187 |
| Mm00441941_m1 | Tfrc | transferrin receptor | 66 |
| Mm00447333_m1 | Snca | synuclein, alpha | 74 |
| Mm00447485_m1 | Tfam | transcription factor A, mitochondrial | 81 |
| Mm00452592_m1 | Ndufb5 | NADH dehydrogenase (ubiquinone) 1 beta subcomplex, 5 | 83 |
| Mm00482889_m1 | Ank1 | ankyrin 1, erythroid | 62 |
| Mm00506532_m1 | Ift140 | intraflagellar transport 140 homolog (Chlamydomonas) | 89 |
| Mm00514993_m1 | Gch1 | GTP cyclohydrolase 1 | 101 |
| Mm00522438_m1 | Map4k5 | mitogen-activated protein kinase kinase kinase kinase 5 | 63 |
| Mm00451095_m1 | Ncoa4 | nuclear receptor coactivator 4 | 69 |
| Mm00452129_m1 | Sirt3 | sirtuin 3 (*S. cerevisiae*) | 60 |
| Mm00442004_m1 | Tsc2 | tuberous sclerosis 2 | 77 |
| Mm00468177_m1 | E4f1 | E4F transcription factor 1 | 143 |
| Mm00476104_m1 | Phb2 | prohibitin 2 | 102 |
| Mm00490673_m1 | Foxo3a | forkhead box O3a | 57 |
